# Molecular determinants of response to PD-L1 blockade across tumor types

**DOI:** 10.1038/s41467-021-24112-w

**Published:** 2021-06-25

**Authors:** Romain Banchereau, Ning Leng, Oliver Zill, Ethan Sokol, Gengbo Liu, Dean Pavlick, Sophia Maund, Li-Fen Liu, Edward Kadel, Nicole Baldwin, Suchit Jhunjhunwala, Dorothee Nickles, Zoe June Assaf, Daniel Bower, Namrata Patil, Mark McCleland, David Shames, Luciana Molinero, Mahrukh Huseni, Shomyseh Sanjabi, Craig Cummings, Ira Mellman, Sanjeev Mariathasan, Priti Hegde, Thomas Powles

**Affiliations:** 1 Genentech, South San Francisco, CA USA; 2 Foundation Medicine, Cambridge, MA USA; 3 Baylor Institute for Immunology Research, Dallas, TX USA; 4 Foundation Medicine, Cambridge, MA USA; 5 Barts Experimental Cancer Medicine Centre, Barts Cancer Institute, Queen Mary University of London, London, UK

**Keywords:** Cancer genomics, Cancer therapy, Tumour immunology

## Abstract

Immune checkpoint inhibitors targeting the PD-1/PD-L1 axis lead to durable clinical responses in subsets of cancer patients across multiple indications, including non-small cell lung cancer (NSCLC), urothelial carcinoma (UC) and renal cell carcinoma (RCC). Herein, we complement PD-L1 immunohistochemistry (IHC) and tumor mutation burden (TMB) with RNA-seq in 366 patients to identify unifying and indication-specific molecular profiles that can predict response to checkpoint blockade across these tumor types. Multiple machine learning approaches failed to identify a baseline transcriptional signature highly predictive of response across these indications. Signatures described previously for immune checkpoint inhibitors also failed to validate. At the pathway level, significant heterogeneity is observed between indications, in particular within the PD-L1^+^ tumors. mUC and NSCLC are molecularly aligned, with cell cycle and DNA damage repair genes associated with response in PD-L1- tumors. At the gene level, the CDK4/6 inhibitor *CDKN2A* is identified as a significant transcriptional correlate of response, highlighting the association of non-immune pathways to the outcome of checkpoint blockade. This cross-indication analysis reveals molecular heterogeneity between mUC, NSCLC and RCC tumors, suggesting that indication-specific molecular approaches should be prioritized to formulate treatment strategies.

## Abstract (web summary)

PD-L1 immune checkpoint inhibition has been used for several tumour types. Here, the authors use immunohistochemistry, tumour mutation burden and RNA-seq data from 366 patients with different indications to identify molecular signatures of response to atezolizumab and reveal pathway heterogeneity and the involvement of non-immune pathways.

## Introduction

The development of cancer treatments, such as chemotherapy or hormone therapy, has traditionally focused on specific tumor types. More recently, immune checkpoint inhibitors (CPIs) that target the cytotoxic T-lymphocyte-associated protein 4 (CTLA4) and the program cell death protein 1 (PD-1) or its ligand PD-L1 have shown durable clinical responses across various cancer types, including melanoma, non-small cell lung cancer (NSCLC), locally advanced or metastatic urothelial carcinoma (mUC), and renal cell carcinoma (RCC)^1,2^. Nevertheless, only a small subset of patients responds durably to CPIs. To maximize patient benefit and minimize toxicity, new biomarkers are needed to identify responders to CPI monotherapy and to inform combination approaches for nonresponders. However, a single biomarker effective across tumor types remains elusive.

In some tumor types, increased PD-L1 expression on both tumor cells (TC) and tumor-infiltrating immune cells (IC) enriches for patients that may respond^3^. In addition, tumor mutation burden, which varies across indications^4^, has been proposed as an independent biomarker of response to CPI. Presumably, increased mutation rate leads to increased neoantigen load, thus enabling antitumor activity by neoantigen-specific CD8^+^ T cells^5^. A T cell gene expression signature in immune-rich tumors has also been recently associated with better outcome following PD-1 blockade^6^. Furthermore, combining TMB with PD-L1 expression^7^ or CD8^+^ T cell signatures within the tumor microenvironment (TME) enriches for responders to checkpoint blockade. Finally, the approval of pembrolizumab in patients with high levels of microsatellite instability, which results from impaired DNA mismatch repair, demonstrated the existence of a pan-tumor biomarker in a very small subset of patients. However, most patients with PD-L1^+^ and/or TMB^high^ tumors do not respond to treatment, and some responders harbor both PD-L1^−^ and TMB^low^ tumors. These findings highlight the need for more reliable biomarkers to predict response and primary resistance to CPI.

In this study, we complement PD-L1 expression and TMB with transcriptional profiling by RNA-seq in 366 patients to identify molecular programs associated with response to CPI in three tumor types that are responsive to the anti-PD-L1 monoclonal antibody atezolizumab.

## Results

### Patient clinical profiles

We analyzed archived tissue from three atezolizumab monotherapy studies, including 208 patients with locally advanced or mUC (IMvigor210 (ref. ^8^), NCT02108652), 81 patients with locally advanced or metastatic NSCLC (POPLAR^9^, NCT01903993), and 77 patients with untreated advanced/metastatic RCC (IMmotion150 (ref. ^10^), NCT01984242; Supplementary Fig. 1a). Patient characteristics are described in Supplementary Data 1. Objective response rate (ORR) was assessed by the Response Evaluation Criteria In Solid Tumors (RECIST) v1.1. Patient outcome was characterized as response (complete response: CR/Partial response: PR) or nonresponse (stable disease: SD/progressive disease: PD). No significant difference in ORR distribution was observed among the three indications. ORR was 21.6% (45/208), 13.6% (11/81), and 19.5% (15/77) in mUC, NSCLC, and RCC respectively (Supplementary Fig. 1b and Supplementary Data 1).

### PD-L1 IHC and tumor mutation burden

**Fig. 1:**
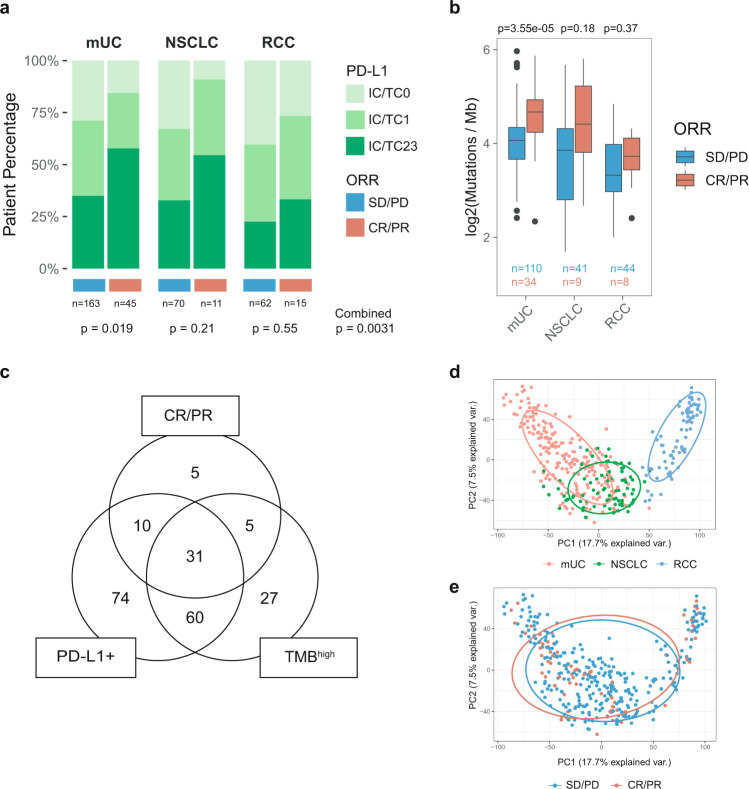
PD-L1, TMB, and global RNA-seq profiles in mUC, NSCLC, and RCC cohorts. **a** Bar chart representing the distribution of PD-L1 expression on TC and IC by indication and response group. PD-L1 distribution between response groups within and across indications was statistically tested using the two-sided Pearson’s chi-squared test. **b** Boxplot representing TMB by indication and response group. TMB differences within each indication were tested using the non-corrected two-sided Wilcoxon rank-sum test. The center of the boxplot represents the median. The lower and upper hinges correspond to the first and third quartiles. The upper whisker extends from the hinge to the largest value no further than 1.5 × IQR (interquartile range) from the hinge. The lower whisker extends from the hinge to the smallest value at most 1.5 × IQR of the hinge. **c** Venn diagram representing the overlap between responders, PD-L1^+^ patients and TMB^high^ patients. **d** Two-dimensional scatter plot representing sample distribution according to the first two components obtained from principal component analysis (PCA) of the complete RNA-seq-evaluable dataset (*n* = 366) based on the 16,581 genes used for analysis. Dots are colored by indication and ellipses capture all samples within one standard deviation of the mean per normal probability statistics (68%). **e** Same as **d**, colored by response group.

We first assessed the distribution of the previously described biomarkers PD-L1 and TMB across indications. A total of 366 pretreatment tumors were assessed for PD-L1 expression on TC and IC by immunohistochemistry (IHC). Tumors were consistently defined as PD-L1^+^ if ≥ 1% of IC or TC stained positively for PD-L1, enabling cross-trial comparison. 74.0% (154/208) of mUC, 70.4% (57/81) of NSCLC, and 62.3% (48/77) of RCC tumors were PD-L1^+^ (Supplementary Fig. 1c and Supplementary Data 1). Across indications, responders exhibited increased proportions of PD-L1^+^ tumors (*p* = 0.0031; Fig. 1a). This inclusive PD-L1 scoring scheme yielded high sensitivity (83.1%), but low specificity (32.2%) to detect responders (mUC: 84.4%/28.8%; NSCLC: 90.1%/32.9%; RCC: 73.3%/40.3%), supporting the need for complementary biomarkers to increase accuracy.

TMB, which quantifies somatic mutations in a tumor, was assessed by whole-exome sequencing (WES) in 246 samples (144 mUC, 50 NSCLC, and 52 RCC). Significant differences in TMB were detected among indications, with tumors from mUC (median 17.7 mut./mb) and NSCLC (median 15.5 mut./mb) exhibiting significantly higher TMB than RCC tumors (median 10.9 mut./mb), consistent with observations from The Cancer Genome Atlas (TCGA)^4^ (Supplementary Fig. 1d). TMB was significantly higher in responders in mUC (*p* = 3.55e−05) and a trend was observed in NSCLC (*p* = 0.15). In this cohort, no significant difference in TMB was observed between response groups in RCC (*p* = 0.37)^10^ (Fig. 1b). Samples were further classified as TMB^high^ or TMB^low^ based on the median (16.3 mut./mb). At this cutoff, the overall sensitivity and specificity of TMB to detect responders were 70.6% and 55.4% (mUC: 79.4%/47.3%; NSCLC: 66.7%/56.1%; RCC: 37.5%/75.0%), with highest sensitivity achieved in mUC.

When investigating PD-L1 and TMB in combination, the union of PD-L1^+^ and TMB^high^ tumors included 207 of 246 total assayed tumors (84.1%), 51 of which were from responders (Fig. 1c). Of these, 31 (60.8%) were PD-L1^+^ and TMB^high^, 10 (19.6%%) were PD-L1^+^ only, and 5 (9.8%) were TMB^high^ only. Five additional tumors (9.8%) from responders were both PD-L1^−^ and TMB^low^. Using PD-L1 expression and TMB jointly identified a majority of responders, improving detection sensitivity. However, PD-L1^+^ and/or TMB^high^ tumors also included 161/195 (82.6%) nonresponders, highlighting poor detection specificity at these cutoffs. False positives and negatives were seen across all tumor groups. This suggested multifactorial tumor-specific mechanisms of response and prompted us to identify complementary biomarkers to detect responders across tumor types more accurately.

### Transcriptional landscape of pretreatment tumors

To complement PD-L1 expression and TMB, pretreatment bulk tumor transcriptomes were profiled by RNA-seq. Principal component analysis (PCA) revealed that samples primarily cluster by tumor type (Fig. 1d). Overlap was observed between mUC and NSCLC tumors, while RCC tumors clustered discretely, likely due to a combination of tissue-specific effects and distinct mechanisms of tumorigenesis. This clustering pattern was confirmed in two independent datasets from TCGA and PCD4989g, a phase I basket trial of atezolizumab monotherapy^11^ (Supplementary Fig. 2a). These observations were supported by principal variance component analysis (PVCA), where cancer type explained 35% of the global variance observed in the data (Supplementary Fig. 2b). Response to treatment (ORR) only explained ~1% of the transcriptional variance across the dataset, suggesting subtle and possibly tumor-intrinsic response patterns (Fig. 1e and Supplementary Fig. 2b).

To interpret biological pathways enriched within the three cancer types, we conducted weighted gene co-expression network analysis (WGCNA)^12,13^ across all samples, identifying 61 modules of co-expressed genes. These were annotated according to pathway enrichment analysis from the Reactome^14^ and Ingenuity Pathway Analysis (IPA)^15^ databases, and guilt-by-association based on hierarchical clustering of module-to-module correlation patterns (Supplementary Fig. 3). We applied these modules to characterize the three cancer transcriptomes (Supplementary Fig. 4). In addition, we conducted bulk RNA deconvolution using xCell^16^ (Supplementary Fig. 5). When aggregated by indication, RCC tumors were enriched for T/NK cell cytotoxicity, type II IFN signaling/antigen presentation, angiogenesis, and myeloid inflammation, while NSCLC tumors were enriched in B/plasma cell and type I IFN signatures. mUC tumors were enriched for proliferation and DNA damage repair (DDR) signatures, possibly reflecting high TMB levels observed in this indication. Within indications, some level of heterogeneity could be observed for most of these signatures. xCell deconvolution further highlighted increased memory B cells, plasma cells, and dendritic cells in NSCLC, and increased memory CD8 T cells, NKT, and macrophages in RCC. Deconvolution also revealed increase in transcriptional programs enriched in epithelial cells in mUC and NSCLC, while RCC tumors were enriched in fibroblasts and endothelial programs, representing increased stroma and vasculature (Supplementary Fig. 5a, b).

Overall, while these tumor types exhibit similar response rates to PD-L1 blockade, their unique transcriptional profiles point toward distinct TMEs that are likely to influence the mechanisms of response to checkpoint inhibition.

### Performance of transcriptional predictors of response to PD-L1 blockade

**Fig. 2:**
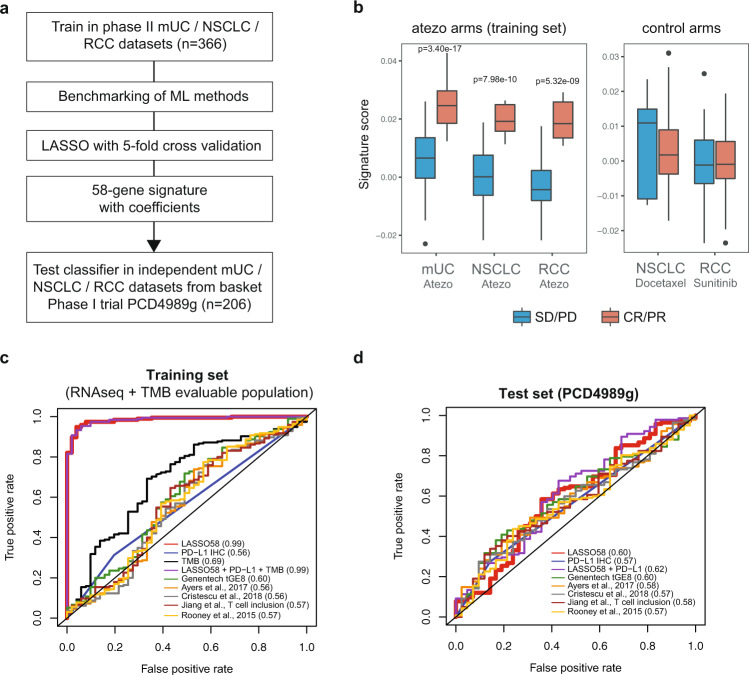
Machine learning to identify a transcriptional signature of response to PD-L1 blockade. **a** Flowchart depicting the approach to identify the signature. **b** Left panel, bar chart representing the signature score by indication and response group within the RNA-seq and TMB-evaluable population in the atezolizumab arms of the training set. *n* = 144 mUC, *n* = 50 NSCLC, and *n* = 52 RCC biologically independent samples were examined. Right panel, bar chart representing the signature score in the control arms of POPLAR (NSCLC, docetaxel arm) and IMmotion150 (RCC, sunitinib arm) clinical trials. *n* = 75 NSCLC and *n* = 85 RCC biologically independent samples were examined. The center of the boxplots represents the median. The lower and upper hinges correspond to the first and third quartiles. The upper whisker extends from the hinge to the largest value no further than 1.5 × IQR (interquartile range) from the hinge. The lower whisker extends from the hinge to the smallest value at most 1.5 × IQR of the hinge. *P* values were calculated using the non-corrected two-sided Wilcoxon rank-sum test. **c** ROC curves for the 58-gene signature, PD-L1, TMB, and external signatures in the RNA-seq/TMB-evaluable population. AUC values are displayed in parenthesis. **d** Same as **c** for the independent phase I PCD4989g test set.

We then applied machine learning to identify a unifying transcriptional signature that could predict response to atezolizumab and complement PD-L1 expression and TMB. Training was conducted in the 366 phase II trial samples. Transcriptomes from 206 independent samples from mUC (*n* = 94), NSCLC (*n* = 54), and RCC (*n* = 58) patients treated with atezolizumab from PCD4989g were used for validation. The least absolute shrinkage and selection operator (LASSO^17^) method combined with fivefold cross-validation (Fig. 2a) yielded a weighted 58-gene signature (Supplementary Data 2) that could segregate responders from nonresponders in the training set (Fig. 2b, left panel). In the chemotherapy (docetaxel) arm from POPLAR (NSCLC) and the tyrosine kinase inhibitor (sunitinib) arm from IMmotion150 (RCC), no significant difference in signature levels was observed between responders and nonresponders, suggesting a predictive value for this signature for response to atezolizumab (Fig. 2b, right panel).

To assess the capacity of this signature to predict responders to atezolizumab monotherapy, we derived receiver operating characteristic (ROC) curves in both training and test sets. We also included ROC curves for PD-L1, TMB, and transcriptional signatures from several previously published studies^6,18–21^. In the training set, our signature demonstrated high accuracy (red curve, AUC = 0.99) in the 246 samples evaluated for both RNA-seq and TMB (Fig. 2c). Both TMB (black curve, AUC = 0.69) and PD-L1 (blue curve, AUC = 0.60) exhibited lower AUC. No synergy was observed between RNA-seq, PD-L1, and TMB, suggesting that RNA-seq alone can recapitulate the biologies underlying PD-L1 expression and TMB. In addition, we tested signatures derived from five previous studies, which were globally enriched for genes involved in IFN-γ and cytotoxic T cell signaling. These signatures exhibited poor predictive performance in our training set, with a maximum AUC of 0.60. We then applied our signature in an independent set of mUC, NSCLC, and RCC tumors treated with atezolizumab (Fig. 2d) in the context of PCD4989g, a phase I basket clinical trial. In this cohort, all signatures tested demonstrated low capacity to predict ORR, including our 58-gene signature, with AUCs <0.65. We benchmarked several other machine learning algorithms on our training and test sets, including random forest, support vector machine, RIDGE, quadratic discriminant analysis, and gradient boosting (Supplementary Fig. 6), which all yielded similar results. These findings suggest that while machine learning methods can identify highly accurate signatures using cross-validation in single datasets, validation in independent datasets remains challenging and potentially confounded by low sample size, heterogeneity of response mechanisms between indications, as well as clinical differences between cohorts.

### Transcriptional correlates of PD-L1 expression and TMB

We next probed into the biological pathways associated with response to atezolizumab both across and within indications. To do so, we developed a model including response, tumor type, PD-L1 expression, and TMB. We first identified genes associated with PD-L1 expression. A total of 1325 genes were overexpressed and 463 genes were underexpressed (FDR-adjusted *p* value < 0.1, absolute log2 fold change ≥ 0.25) in PD-L1^+^ vs. PD-L1^−^ tumors (Supplementary Data 3). Overexpressed genes were enriched for immune signatures, including IFN-γ-induced chemokines (*CXCL9*, *CXCL10*, *CXCL11*, and *CXCL13*), checkpoints (*CTLA4* and *IDO1*), as well as genes encoding for cytotoxicity (*GZMA*, *GZMB*, *GZMH*, *GZMK*, and *PRF1*) and B/plasma cell biology (*CD79A*, *JCHAIN*, *IGLL5*, *MZB1*, *TNFRSF13B*, and *BLK*). We further analyzed biological pathway enrichment among genes correlated with PD-L1 expression in all tumors combined (Supplementary Fig. 7, upper panel) or by indication (Supplementary Fig. 7, lower panel) using Q-Gen^22^, a generalization of quantitative set analysis for gene expression (QuSAGE)^23^ that integrates fixed and random effects from generalized linear models. PD-L1^+^ tumors showed an increase in type I/II IFN signaling and lymphoid signatures across tumor groups, as described previously within single tumor types, while PD-L1^−^ tumors showed increased in metabolic pathways and zinc finger proteins. However, indication-specific associations of other pathways, such as myeloid inflammation (RCC), cell cycle/proliferation (mUC), and metabolism (RCC) were also observed, suggesting that regulation of PD-L1 expression in the TME is complex and indication-dependent.

Similarly, we compared the transcriptomes of TMB^high^ and TMB^low^ tumors. A total of 165 genes were overexpressed and 121 genes underexpressed (FDR-adjusted *p* value < 0.1, absolute log2 fold change ≥ 0.25) in TMB^high^ tumors, demonstrating weaker association between TMB and transcription than PD-L1 (Supplementary Data 4), as confirmed by PVCA (Supplementary Fig. 2). The most significantly overexpressed gene in TMB^high^ tumors was E2F1 (*p* = 0.0098), which encodes a transcription factor essential for cell cycle, DDR, and tumor suppression regulation^24^. Q-Gen analysis across tumor types revealed limited enrichment and consistency across tumor types of signatures according to TMB status, suggesting multiple biological processes that could potentially lead to high TMB (Supplementary Fig. 8).

These data demonstrate that, of the biomarkers measured, PD-L1 expression contributes the most to the transcriptional variance observed by RNA-seq in bulk tumors, and that genes associated with PD-L1 levels represent a surrogate signature of the immune infiltrate across tumor types. Transcriptional correlates of PD-L1 and TMB are mostly distinct, supporting nonoverlapping characteristics between these two biomarkers.

### Transcriptional correlates of response to PD-L1 blockade

Next, we explored associations between gene expression and response to atezolizumab. Because of the limited effect of TMB on bulk tumor transcriptomes, TMB was removed from the model to maximize sample size and power. Integrating PD-L1 expression and indication as covariates, only 32 genes were overexpressed and 59 genes were underexpressed (FDR-adjusted *p* value < 0.1, absolute log2 fold change ≥ 0.25) in responders, revealing few transcriptional correlates of ORR across indications at the gene level (Supplementary Data 5).

**Fig. 3:**
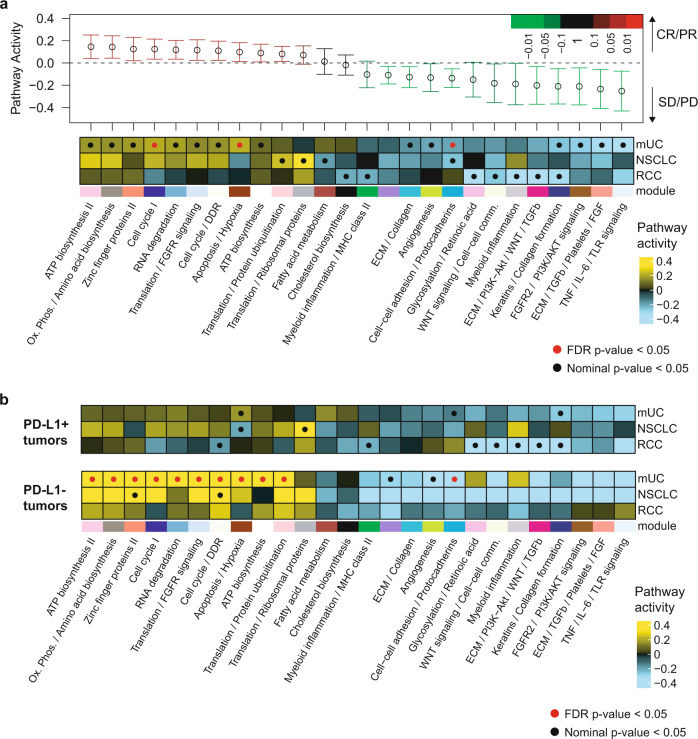
Transcriptional correlates of response to PD-L1 blockade across indications. **a** In all tumors combined. Upper panel, Forest plot representing the pathway activity of WGCNA modules significantly enriched by Q-Gen analysis between responders and nonresponders across the combined cohorts or on an individual cohort basis. The pathway-level model contrasts responders and nonresponders, including indication as a covariate in the cross-indication analysis. The 20 modules detected as significant (*p* < 0.05) in any of the four models conducted are included in the display. Modules are ordered from left to right according to pathway activity across indications. Error bars represent the 95% confidence interval. Lower panel, heatmap representing per-indication pathway activity for modules selected from the combined cross-indication and per-indication analyses. Module enrichment significance is highlighted as a red dot (FDR-corrected *p* < 0.05) or a black dot (nominal *p* < 0.05). *n* = 208 mUC, *n* = 81 NSCLC, and *n* = 77 RCC biologically independent samples were examined. **b** Same as **a** for PD-L1^+^ (upper heatmap) and PD-L1^−^ (lower heatmap) tumors separately.

Module enrichment analysis was initially performed on all samples across indications. (Fig. 3a, upper panel) or within each indication (Fig. 3a, heatmap). While no single module was significantly associated with response, positively or negatively, across indications, mUC and NSCLC tumors displayed more similarities than RCC tumors, consistent with their higher transcriptional similarity overall (Fig. 1d and Supplementary Fig. 2a). In the combined analysis (Fig. 3a, upper panel), responsive tumors were enriched in ATP biosynthesis and oxidative phosphorylation. Cell cycle/DDR was also enriched in mUC and NSCLC tumors, possibly reflecting the association between increased TMB and response to PD-L1 blockade. Conversely, modules related to tumor biology (WNT and PI3K-Akt) and stromal biology (TGF-β, collagens, and extracellular matrix) were enriched in nonresponders, as we previously reported in mUC^18^. Nonresponsive RCC tumors were also enriched for metabolic and myeloid inflammation signals, which appeared distinct from mUC and NSCLC^10^.

Subsequent analyses were conducted separately in PD-L1^+^ and PD-L1^−^ tumors, revealing significant differences in transcriptional correlates of responses according to PD-L1 status. Within the 259 PD-L1^+^ tumors, no single module was significantly associated with ORR across individual indications. In PD-L1^+^ mUC tumors, only apoptosis signals associated with response. PD-L1^+^ NSCLC were dominated by translation pathways. In PD-L1^+^ RCC tumors, myeloid inflammation, WNT signaling and collagen formation negatively associated with ORR (Fig. 3b, upper panel). Interestingly, more uniform transcriptional signals were observed across indications in the 107 PD-L1^−^ tumors. Cell cycle/DDR positively associated with response across PD-L1^−^ tumors (Fig. 3b, lower panel). While this suggests the contribution of high TMB to response in this setting, the correlation between the DDR signature and TMB was modest (Pearson *R* = 0.14, Supplementary Data 6).

Finally, we analyzed ORR associations with cell populations deconvoluted with xCell (Supplementary Fig. 9). No cell population demonstrated a consistent association with ORR across indications. Pro B cell, pDC, and basophils were increased in responders in mUC, while DC, monocytes, and NKT cells were increased in responders in NSCLC. Only central memory CD8^+^ T cells were increased in responders in RCC.

These analyses provide insight into the complexity of mechanisms of response to PD-L1 blockade, highlighting heterogeneity in pathways associated with response to atezolizumab across tumor types, especially within PD-L1^+^ tumors. Transcriptional features associated with response appear multifactorial and are in part determined by the histological features of the tumor and possibly by the various cell populations that express PD-L1.

### Increased CDKN2A activity in responders to PD-L1 blockade

**Fig. 4:**
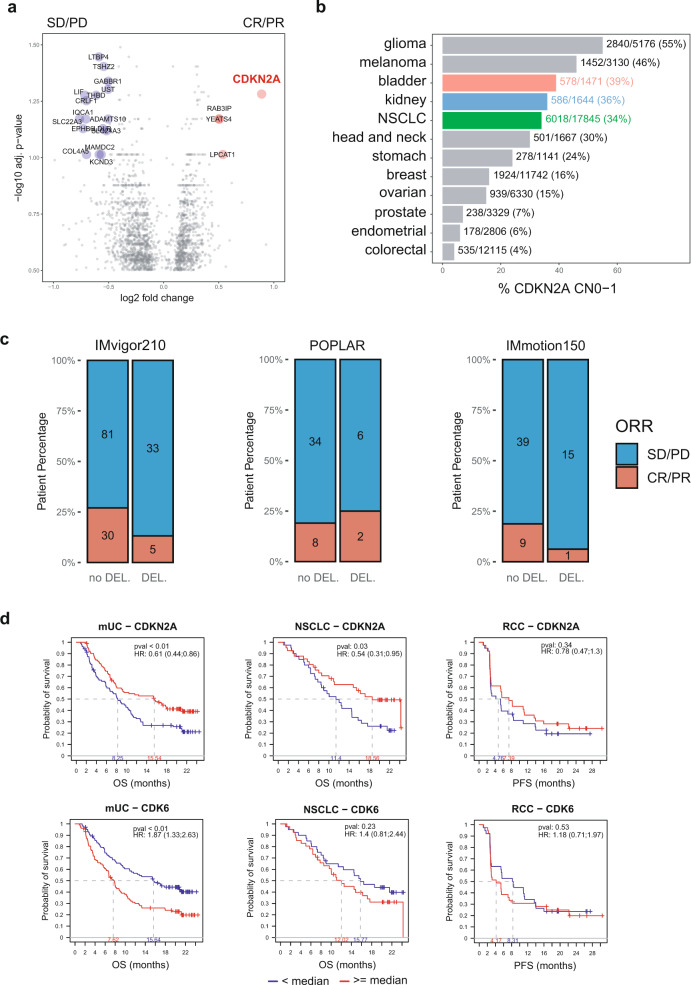
CDK4/6 inhibition associates with increased response to PD-L1 blockade. **a** Volcano plot representing the genes differentially expressed between responders and nonresponders. The gene-level linear model contrasts responders (CR/PR) and nonresponders (SD/PD), including indication and PD-L1 expression as covariates. Genes significantly upregulated or downregulated after Benjamini–Hochberg correction (*p* < 0.1) and absolute log2 fold change ≥  0.5 are colored in red and blue, respectively. **b** Horizontal bar chart representing the percent of patients exhibiting partial (CN1) or complete copy-number deletion (CN0) of the *CDKN2A* locus across the Foundation Medicine database (*n* = 97,811 after QC) for selected indications. The ratio and percentage of patients with *CDKN2A* loss within each ontology is represented on the right of each bar. **c** Response rate by *CDKN2A* deletion status. Bar charts represent the proportion of responders to nonresponders by *CDKN2A* deletion status (no DEL: copy-number ≥ 2; DEL: copy-number < 2). *P* values were calculated using the two-sided Pearson’s chi-squared test. **d** Overall survival (OS) for mUC and NSCLC cohorts and progression-free survival (PFS) for the RCC cohort, split by transcriptional expression of *CDKN2A* (top) or *CDK6* (bottom). Transcription level is defined as high (≥median, red) or low (<median, blue) within each indication.

Among the genes associated with response to PD-L1 blockade, CDKN2A was most upregulated (log2-FC = 0.89, *p* = 0.05; Fig. 4a). *CDKN2A* encodes for p16(INK4A), an endogenous inhibitor of the cyclin-dependent kinases CDK4 and CDK6, which prevents G1/S phase transition and induces cell senescence^25^. *CDKN2A* undergoes copy-number loss in many tumor types. To assess the prevalence of *CDKN2A* deletions in mUC, NSCLC, and RCC, we queried the Foundation Medicine clinical database, including 140,288 patient tumor samples, for the prevalence of patients with partial (one copy, CN1) or complete (zero copy, CN0) *CDKN2A* deletion. Several indications exhibited frequent *CDKN2A* loss (Fig. 4b), including bladder (39%), RCC (36%), and NSCL cancers (34%).

We then assessed the association between *CDKN2A* copy-number alterations and response to PD-L1 blockade in our cohorts, focusing on tumors profiled both by WES and RNA-seq. Using Sequenza^26^, we determined copy-number alterations in *CDKN2A* across samples evaluated by WES. Decreased response to atezolizumab in tumors with CDKN2A loss was observed in mUC and RCC, yet this association did not reach statistical significance, possibly due to low sample size (Fig. 4c). Because *CDKN2A* and *CD274* (encoding PD-L1) are both encoded on the p arm of chromosome 9 (chr9p), we also examined the co-occurrence of *CDKN2A* and *CD274* deletions in the FMI database. This analysis demonstrated that *CD274* deep deletions are exceedingly rare in this large database, with only 0.04% (62/140,288) of samples harboring a *CD274* homozygous deletion. While *CDKN2A* deletions commonly co-occur with deletion of *CDKN2B* (89% of *CDKN2A* events), only a small fraction of *CDKN2A* deletions are accompanied by deletion in *CD274* (0.2%). We also identified shallow deletions in the chr9p arm in WES data from the three trials, and tested the effect of these deletions on *CD274* and *CDKN2A* expression and correlation (Supplementary Fig. 10). Shallow chr9p deletions were observed in 14.9%, 19.5%, and 19.8% of POPLAR, IMvigor210, and IMmotion150 tumors, respectively, highlighting a similar chr9p deletion rate across indications. We did not find robust associations between chr9p deletion status and *CD274* or *CDKN2A* expression, which may be due to the contribution of non-tumoral cell populations to the bulk transcriptional expression of these genes. Finally, we analyzed associations between the transcriptional levels of *CDKN2A* and its two targets *CDK4* and *CDK6* with overall survival (OS) for mUC and NSCLC, and progression-free survival (PFS) in RCC (Fig. 4d). Increased *CDKN2A* associated with improved OS in mUC (HR = 0.61, *p* < 0.01) and NSCLC (HR = 0.54, *p* = 0.03), and trended to improved PFS in RCC (HR 0.78, *p* = 0.34). Conversely, increased *CDK6* significantly associated with decreased OS in mUC (HR: 1.87, *p* < 0.01) and showed a trend toward lower OS in NSCLC (HR = 1.40, *p* = 0.23), and lower PFS in RCC (HR = 1.2, *p* = 0.5). No differences were observed for *CDK4*.

Several CDK4/6 inhibitors (CDK4/6i), which are pharmacological equivalents of p16(INK4A), are currently used in the clinic to treat hormone receptor-positive breast cancer^27^. Altogether, these observations, along with several recent preclinical studies combining CDK4/6i with CPI^28–32^, suggest that biomarker-selectable subsets of patients from mUC, NSCLC, and RCC may also benefit from combination therapy targeting both PD-(L)1 and CDK4/6 axes simultaneously.

## Discussion

Biomarker discovery in the context of clinical checkpoint inhibition is in its infancy. PD-L1 expression and TMB are currently the only actionable biomarkers in some indications. Previous studies have focused on small cohorts in single indications or large cohorts with limited clinical outcome information. Herein, we performed a unified molecular analysis within prospective studies in >800 tumors, including 572 patients on atezolizumab monotherapy, to analyze common and specific molecular programs in three tumor types responsive to PD-L1 blockade. We characterized a heterogeneous transcriptional landscape of early response to therapy, defined by ORR, across tumor types, which could not be systematically recapitulated by a single baseline transcriptional signature through advanced machine learning. These tumor-specific observations are being mirrored in the clinic, where treatments are showing tumor-specific efficacy and distinct associations with biomarkers^10,33,34^. It is possible that the relatively low size of the training set, as well as the clinical differences between training (phase II trials) and test (phase I basket trial) sets impact our findings. Nevertheless, we identified the CDK4/6 inhibition axis as a potential correlate of response in mUC and RCC tumors treated with atezolizumab, suggesting that subsets of tumors may share mechanisms of response and resistance to cancer immunotherapy.

While PD-L1 expression and high TMB were associated with increased ORR, as recently described by Cristescu et al. in the context of PD-1 inhibition with pembrolizumab^19^, these biomarkers exhibited low specificity and therefore limited accuracy to identify responders to atezolizumab. In addition, when combining these biomarkers, 10% of responders were both PD-L1^−^ and TMB^low^, suggesting the existence of independent mechanisms of response to PD-L1 blockade. Ayers et al. recently described a 19-gene IFN-γ signature measured by a targeted assay, which associated with clinical response to pembrolizumab across nine tumors^6^. The restricted expression of PD-1 on T cells and subsets of B cells supports this IFN-γ signature as an effective biomarker, especially in inflamed tumors. This signature, along with our tGE8 signature and others recently described^19–21^, failed to accurately identify responders to PD-L1 blockade in both our training and test cohorts. In these analyses, we have calculated median signature expression scores, as genes from these signatures tend to be co-expressed, but it is possible that fine-tuning score calculation, by weighting specific genes for example, may improve their performance. These findings may also be caveated by the sample size and makeup of our training and test sets, which may account for differences observed across. Further machine learning efforts are warranted in larger cohorts of patients treated with CPIs.

Our unbiased transcriptomic approach, combining machine learning and linear modeling, brings additional insights into the biological pathways associated with response to PD-L1 blockade, both in the context of PD-L1^+^ and PD-L1^−^ tumors. Our data highlight the association of senescence control, ATP biosynthesis, translation, zinc finger protein activity, and myeloid inflammation with response to atezolizumab. They also revealed overlap between mUC and NSCLC tumors, and segregation of RCC tumors. Using a dimension-reducing approach, we identified modules of co-expressed genes that capture many components of the TME. While IFN-γ and antigen presentation were associated with response and myeloid inflammatory signatures were associated with lack thereof, no module was strongly associated with outcome across tumor types. Further module analysis in PD-L1^+^ tumors failed to show universal signatures of response, suggesting significant heterogeneity in mechanism of response. In PD-L1^−^ tumors, cell cycle and DDR appeared as the single unifying mechanism of response across tumors, which may reflect increased TMB and neoantigen burden, albeit poor correlation was observed between these signatures and TMB. The identification of consistent subsets of PD-L1 negative tumors that respond to therapy highlight the importance of multiple biomarker analysis to improve accuracy. Overall, our data suggest that no single transcriptional program associates with early response to atezolizumab across tumor types and PD-L1 expression phenotypes. The investigation of predictive transcriptional signatures for other end points, such as OS are warranted in larger datasets.

When analyzing correlates of response at the gene level, *CDKN2A*, a tumor suppressor gene that inhibits CDK4/6 activity and is frequently deleted across cancers, was the strongest correlate of response to atezolizumab. Several studies have demonstrated synergy between CDK4/6 and PD-(L)1 inhibitors in preclinical models^28–30^. The potential mechanisms of synergy include (i) increase in type III IFN following ERV reactivation and increase in MHC I presentation in TC^28^; (ii) increase in PD-L1 expression following CDK4 blockade^31^; and (iii) decrease in regulatory T cell proliferation. We observed an inverse association between *CDKN2A* and *CDK6* transcription and a trend toward lower response in tumors that exhibited copy-number loss in *CDKN2A*. Because non-tumors cells present in the TME can contribute to *CDKN2A* mRNA, it remains challenging to interpret bulk transcriptional data, and single-cell RNA-seq profiling will be needed to dissect population-specific transcription of this gene. A recent study leveraging single-cell RNA-seq in tumors from melanoma patients treated with anti-PD-1 identified the CDK4/6 axis as a tumor-intrinsic resistance mechanism^32^. Another study^35^ identified co-occurrence of *CDKN2A* and *JAK2* chromosomal loss, leading to reduced IFN-γ activity, thereby proposing an explanation for reduced response to checkpoint inhibition. We queried Foundation Medicine’s database of >140,000 solid tumors to examine co-occurrence of *JAK2*, and *CDKN2A/B* alterations in adult solid tumors. Of the 20,014 samples with a *CDKN2A/B* deletion, only 73 had a co-occurring *JAK2* deletion (0.4%), revealing a low nominal rate of co-occurrence of *JAK2* alterations and *CDKN2A* deletions in this database. Interestingly, a recent study has identified benefit from checkpoint blockade in a small cohort of melanoma patients with germline *CDKN2A* mutations^36^, suggesting a different effect from germline and somatic mutations. This should be confirmed in larger randomized settings and expanded to other indications.

This cross-indication analysis, combining existing biomarkers with RNA-seq in 572 patients treated with atezolizumab across three indications, revealed significant molecular heterogeneity between mUC, NSCLC, and RCC tumors. Machine learning did not identify a unifying transcriptional signature predictive of ORR. Multiple factors appear to determine response to checkpoint blockade, which are in part tumor type-dependent, highlighting the difficulty of biomarker development in this field. While universal biomarkers of response to PD-(L)1 blockade may exist, it will be essential to consider indication-specific molecular contexts to formulate the next generation of combination therapies.

## Methods

### Study design, patient cohorts, PD-L1 testing, and response assessment

A total of 366 patients from three phase II clinical trials of atezolizumab were selected for analysis. These included 208 patients from a single-arm phase II clinical trial of atezolizumab in second-line mUC (IMvigor210, cohort 2 (ref. ^8^), NCT02108652), 81 patients from a randomized phase II clinical trial of atezolizumab vs. docetaxel in second-line NSCLC (POPLAR^9^, NCT01903993), and 77 patients from a randomized phase II clinical trial of atezolizumab vs. atezolizumab + bevacizumab vs. sunitinib in front-line RCC (IMmotion150 (ref. ^10^), NCT01984242). We complied with all relevant ethical regulations for work with human participants, and informed consent was obtained from all patients. The protocol was approved by the institutional review boards or independent ethics committees at each participating center. PD-L1 expression was tested by IHC as described below. Response was assessed by RECIST v1.1. Patients who did not reach an evaluable RECIST score as defined by CR/PR/SD/PD were excluded from the study. For validation, 94 patients with mUC, 54 patients with NSCLC, and 58 patients with RCC were selected from the phase I basket trial of atezolizumab PCD4989g^11^ (NCT01375842).

### PD-L1 immunohistochemistry and categorization

Prescreening biopsies were collected from archived paraffin-embedded tissue (FFPE). Patients were required to have tissue sent to the central laboratory before study entry. Samples were processed at the time of screening. FFPE tumor tissue was stained prospectively for PD-L1 by IHC using a proprietary diagnostic anti-human PD-L1 monoclonal antibody (SP142, Ventana, cat no: 740–4859, diluted at 7 µg/mL). Samples were scored for PD-L1 expression separately on TC and tumor-infiltrating IC. For TC, specimens were scored as PD-L1 TC0, TC1, TC2, or TC3 if <1%, ≥1% but <5%, ≥5% but <50%, or ≥50% of TC were PD-L1 positive, respectively. For IC, specimens were scored as PD-L1 IC0, IC1, IC2, or IC3 if specimens were scored as <1%, ≥1% but <5%, ≥5% but <10%, or ≥10% of IC were PD-L1 positive, respectively. PD-L1 scores in patients with multiple specimens from different time points or samples were based on the highest score. For analytical purposes, samples were categorized as PD-L1^+^ if any level of PD-L1 expression (1, 2, or 3) was detected on either IC or TC.

### Nucleic acid sample preparation

The pathologic diagnosis of each case was confirmed by hematoxylin and eosin (H&E) stained slides and nucleic acid extraction was conducted for all samples that contained a minimum of 20% TC. H&E images were marked for macro-dissection by a pathologist. RNA (High Pure FFPET RNA Isolation Kit, Roche) and DNA (QIAamp DNA FFPE Tissue Kit, QIAgen) were then extracted from the macro-dissected sections. Whole-transcriptome profiles were generated using TruSeq RNA Access technology (Illumina®).

### Comprehensive genomic profiling (CGP) by FoundationOne

CGP was carried out in a Clinical Laboratory Improvement Amendments-certified, College of American Pathologists-accredited laboratory (Foundation Medicine Inc., Cambridge, MA, USA) on all-comers during the course of routine clinical care. Approval was obtained from the Western Institutional Review Board (Protocol No. 20152817). Hybrid capture was carried out for all coding exons from up to 395 cancer-related genes plus select introns from up to 31 genes frequently rearranged in cancer. We assessed all classes of genomic alterations, including short variant, copy number, and rearrangement alterations. Briefly, DNA was extracted from FFPE specimens, of which at least 50 ng underwent whole-genome shotgun library construction and hybridization-based capture. Using the Illumina platform, hybrid-capture-selected libraries were sequenced to high uniform depth (>500×). Substitutions were called using a Bayesian approach. Insertions/deletions were called by de novo local assembly with a de Bruijn approach. Deep deletions (CN = 0) were called from copy-number profiles generated through the statistical fitting of normalized coverage from exons and genome-wide SNPs. Rearrangements were detected through the analysis of chimeric read pairs^37^. Shallow copy-number loss (CN = 1) was called using similar methodology to arm-level calling^38^. Normalized coverage data for exonic, intronic, and SNP targets accounting for stromal admixture were plotted on a logarithmic scale and minor allele SNP frequencies were concordantly plotted. Custom circular binary segmentation further clustered targets and minor allele SNPs to define upper and lower bounds of genomic segments. Signal-to-noise ratios for each segment were used to determine whether it was gained or lost. The sum of those segment sizes determined the fraction of each segment gained or lost. *CDKN2A* was considered lost if >50% of the NM_058197 locus was significantly decreased in log ratio. Analysis was limited to samples with a QC passed copy-number profile (*n* = 97,811). CNA prevalence was aggregated by indication.

### Whole-exome sequencing and tumor mutation burden quantification

Sequencing data were processed to obtain high quality reads and alignments were performed using GSNAP to human reference genome GRCh38, using HTSeqGenie (version 4.0.1). Duplicate reads were marked using PICARD. GSNAP arguments for RNA-seq alignments: -M 2 -n 10 -B 2 -i 1 -N 1 -w 200000 -E 1–pairmax-rna=200000–clip-overlap; GSNAP arguments for exome alignments: -M 2 -n 10 -B 2 -i 1–pairmax-dna=1000–terminal-threshold=1000–gmap-mode=none–clip-overlap. Somatic mutations were called using Lofreq <version 2.1.2> and Strelka <version 1.0.14>. To assess contamination levels and possible mismatch between tumor and whole blood control samples, bam files were used to generate pileup summaries with GATK (v4.0.8.1) tools GetPileupSummaries (gatk GetPileupSummaries -I in.bam -V snp.vcf.gz -L snp.vcf.gz -O out.pileups.table) and CalculateContamination (gatk CalculateContamination -I out.pileups.table -O out.contamination.txt). For tumor samples, the tool was run with the matched normal option (CalculateContamination -I tumor.pileups.table -matched normal.pileups.table -O tumor.contamination.txt). The snp.vcf.gz used is a VCF of common germline variants, which can be acquired using the GATK best-practices resource bundle (https://software.broadinstitute.org/gatk/download/bundle) to download germline variants from gnomad, and subsetting to biallelic SNP sites with >1% allele frequencies. The impact of somatic mutations on proteins was determined using variant effect predictor. Mutations in Entrez genes with these effects were retained: truncation (frameshifts and stop gains), deleterious.missense (non-synonymous and predicted deleterious by Condel), inframe.indel, or missense (non-synonymous but predicted by Condel to be neutral). Expressed mutations were identified using the criteria that at least two RNA-seq reads containing the mutation align at the mutant locus.

### Copy-number alteration profiling by WES

WES reads for all tumor/normal pairs were mapped to the hg38 genome reference using a NGS data analysis pipeline comprised of standard GATK tools for read preprocessing and GSNAP for alignments^39^. Allele-specific copy-number alterations and tumor purity estimates were determined from the resulting BAM files, using Sequenza^26^ version 2.2.0.9000 with default parameters in R version 3.4.2. Copy-number alterations were categorized for each segment as “AMP”, “ampLOH”, “cnLOH”, “DELLOH”, “HOMDEL”, and “NORMAL”, which were defined as follows: NORMAL, total copy number of 2, 1 A allele, 1 B allele; HOMDEL, total copy number of 0, DELLOH, total copy number of 1 with either A or B allele having copy number of 0; cnLOH, total copy number of 2 with either A or B allele having copy number of 0; AMPLOH, total copy number >2 with either A or B allele having copy number of 0; AMP, total copy number >2 with both A or B alleles having copy number of at least 1. Copy-number deletions for *CDKN2A* and other specific genes were called by first identifying a single segment per sample spanning the gene of interest, and then taking only those with “DELLOH” or “HOMDEL” designations. If no single segment could be identified spanning the gene of interest, the sample was excluded from the analysis.

### RNA-seq data processing and normalization

Whole-transcriptome profiles were generated for 817 patients, as previously described^18^. FASTQ files from the three phase II trials and the phase I basket trial PCD4989g were processed using Genentech’s internal stranded pipeline. Raw counts were then normalized using transcript-per-million and log2-transformed.

### Principal component analysis and principal variance component analysis

PCA was conducted on the 16,581 genes detected within the dataset using the *prcomp()* function. PCA analysis was also conducted on mUC, NSCLC, and RCC cohorts from TCGA and the phase I basket trial of atezolizumab PCD4989g. PVCA is a hybrid method that combined PCA and VCA to quantify the contribution of known variables to the global variance observed in the transcriptional data. PVCA analysis was conducted using the *pvca* package (v1.18.0. Bioconductor). The threshold was set to 0.5. The mixed linear model was fit on the following factors: indication, PD-L1 expression, TMB, and ORR.

### Prognostic signature identification and validation

An ensemble model with LASSO^17^-based learner was used. Ensemble models are known to be more robust and less affected by initial seeds. This regression analysis method concurrently performs variable selection and regularization to improve accuracy. To build the base learners, 1000 LASSO models were built using different seeds. In each model, hyper-parameter selection was conducted by fivefold cross-validation. The final ensemble model was built by taking average of the coefficient of the 1000 LASSO models. In more details, in each LASSO model, each gene is assigned a coefficient (if a gene is not selected by this model, the coefficient will be 0). A gene’s coefficient in the final ensemble model is then obtained by taking average of its coefficient in each individual LASSO model. The R package glmnet (v2.0-13) was used^40^.

### Differential gene expression association with PD-L1 IHC, TMB, and ORR

To identify genes associated with PD-L1 expression, TMB, or ORR, we developed a generalized linear model using the *limma* R package (v3.46.0, Bioconductor), which included these three variables, as well as cancer type as covariates (~ORR + PD-L1 + TMB + indication). Each variable was categorized: ORR: responders (CR/PR) vs. nonresponders (SD/PD); PD-L1: positive vs. negative; TMB: high vs. low; Indication: mUC/NSCLC/RCC). Because this model could only be applied to the subset of all samples with TMB measurements (*n* = 246) and the limited effect of TMB on transcriptional variance, we developed a second model that did not include TMB as a covariate and could include all 366 samples to maximize power.

### Gene set extraction and annotation

To extract sets of genes co-expressed within the dataset, we applied WGCNA, using the available R package (v1.64-1)^12,13^. A total of 13,863 genes that exhibited a log2 fold change ≥ 1 in at least ten samples were selected and used as input for module extraction. The WGCNA *blockwiseModules()* function was run with the following parameters: *minModuleSize* = 15, *power* = 5, *mergeCutHeight* = 0.15, and *minKMEtoStay* = 0.3. A soft thresholding power of 5 was selected based on the scale independence chart, as described in the WGCNA tutorials. 62 modules were identified. The “gray” module (unassigned genes) was not considered in downstream analyses. The remaining 61 modules were annotated using a combination of human expertise and pathway enrichment analysis leveraging the Reactome, KEGG, and IPA^15^ databases. Reactome pathway enrichment analysis was conducted using the *ReactomePA (v1.22.0)*^41^ package (Bioconductor). Module annotation was further guided using a guilt-by-association approach. Mean *z*-scores for the 61 modules were derived for the 366 patients, as described below. A 61 × 61 Pearson correlation matrix was then obtained using the R *cor()* function and hierarchically clustered (Euclidian distance), using the *ComplexHeatmap*^42^ package (v2.6.2, Bioconductor; Supplementary Fig. 3).

### Module *z*-score computation

Module scores were quantified per sample as the mean of the *z*-score of the genes composing the module across the dataset.

### Gene set enrichment analysis with Q-Gen

Module enrichment in pairwise comparisons for PD-L1^+^ vs. PD-L1^−^ tumors, TMB^high^ vs.TMB^low^ tumors, or responders vs. nonresponders was conducted using the ggen() function from the *QuSAGE* package (v2.12.0, Bioconductor)^22,23^. Q-Gen is a generalization of QuSAGE^23^ that integrates fixed and random effects from generalized linear models.

### Statistics and reproducibility

Unless otherwise stated, all two-group comparisons for continuous variables use the two-sided Wilcoxon rank-sum test (R function wilcox.test). For categorical variables, the two-sided Pearson’s chi-squared test with continuity correction is used. Unless otherwise stated, FDR-corrected *p* values are reported. Measurements were taken from distinct samples. Indication and PD-L1 expression were added as covariates in linear models.

### Reporting summary

Further information on research design is available in the Nature Research Reporting Summary linked to this article.

## Supplementary information

Supplementary Material

Descriptions of Additional Supplementary Files

Supplementary Data 1

Supplementary Data 2

Supplementary Data 3

Supplementary Data 4

Supplementary Data 5

Supplementary Data 6

Reporting Summary

## Data Availability

All raw RNA-seq and whole-exome sequencing data, along with clinical data, are available under restricted access in the European Genome-Phenome Archive under accession number EGAS00001004343. The remaining data are available within the article, Supplementary Information, the accompanying data.rdata file on EGA (including the CDKN2A loss data), or available from the authors upon request.

## Code availability

Code to reproduce the figures is provided as an.rmd template at http://research-pub.gene.com/cross_indication_2021.

## References

[1] Ribas A, Wolchok JD (2018). Cancer immunotherapy using checkpoint blockade. Science.

[2] Sharma P, Allison JP (2015). The future of immune checkpoint therapy. Science.

[3] Havel, J. J., Chowell, D. & Chan, T. A. The evolving landscape of biomarkers for checkpoint inhibitor immunotherapy. *Nat. Rev. Cancer*. 10.1038/s41568-019-0116-x (2019). 10.1038/s41568-019-0116-x PMC6705396 30755690

[4] Alexandrov LB (2013). Signatures of mutational processes in human cancer. Nature.

[5] Rizvi NA (2015). Cancer immunology. Mutational landscape determines sensitivity to PD-1 blockade in non-small cell lung cancer. Science.

[6] Ayers M (2017). IFN-gamma-related mRNA profile predicts clinical response to PD-1 blockade. J. Clin. Investig..

[7] Powles T (2018). Atezolizumab versus chemotherapy in patients with platinum-treated locally advanced or metastatic urothelial carcinoma (IMvigor211): a multicentre, open-label, phase 3 randomised controlled trial. Lancet.

[8] Balar AV (2017). Atezolizumab as first-line treatment in cisplatin-ineligible patients with locally advanced and metastatic urothelial carcinoma: a single-arm, multicentre, phase 2 trial. Lancet.

[9] Fehrenbacher L (2016). Atezolizumab versus docetaxel for patients with previously treated non-small-cell lung cancer (POPLAR): a multicentre, open-label, phase 2 randomised controlled trial. Lancet.

[10] McDermott DF (2018). Clinical activity and molecular correlates of response to atezolizumab alone or in combination with bevacizumab versus sunitinib in renal cell carcinoma. Nat. Med..

[11] Herbst RS (2014). Predictive correlates of response to the anti-PD-L1 antibody MPDL3280A in cancer patients. Nature.

[12] Zhang, B. & Horvath, S. A general framework for weighted gene co-expression network analysis. *Stat. Appl. Genet. Mol. Biol*. **4**, Article17 (2005). 10.2202/1544-6115.1128 16646834

[13] Langfelder P, Horvath S (2008). WGCNA: an R package for weighted correlation network analysis. BMC Bioinformatics.

[14] Fabregat A (2018). The reactome pathway knowledgebase. Nucleic Acids Res..

[15] Kramer A, Green J, Pollard J, Tugendreich S (2014). Causal analysis approaches in Ingenuity Pathway Analysis. Bioinformatics.

[16] Aran D, Hu Z, Butte AJ (2017). xCell: digitally portraying the tissue cellular heterogeneity landscape. Genome Biol..

[17] Tibshirani R (1996). Regression shrinkage and selection via the Lasso. J. R. Stat. Soc. B Methodol..

[18] Mariathasan S (2018). TGFbeta attenuates tumour response to PD-L1 blockade by contributing to exclusion of T cells. Nature.

[19] Cristescu, R. et al. Pan-tumor genomic biomarkers for PD-1 checkpoint blockade-based immunotherapy. *Science* **362**, eaar3593 (2018). 10.1126/science.aar3593 PMC6718162 30309915

[20] Jiang P (2018). Signatures of T cell dysfunction and exclusion predict cancer immunotherapy response. Nat. Med..

[21] Rooney MS, Shukla SA, Wu CJ, Getz G, Hacohen N (2015). Molecular and genetic properties of tumors associated with local immune cytolytic activity. Cell.

[22] Turner JA, Bolen CR, Blankenship DM (2015). Quantitative gene set analysis generalized for repeated measures, confounder adjustment, and continuous covariates. BMC Bioinformatics.

[23] Yaari G, Bolen CR, Thakar J, Kleinstein SH (2013). Quantitative set analysis for gene expression: a method to quantify gene set differential expression including gene-gene correlations. Nucleic Acids Res..

[24] Dimova DK, Dyson NJ (2005). The E2F transcriptional network: old acquaintances with new faces. Oncogene.

[25] Sherr CJ (2001). The INK4a/ARF network in tumour suppression. Nat. Rev. Mol. Cell Biol..

[26] Favero F (2015). Sequenza: allele-specific copy number and mutation profiles from tumor sequencing data. Ann. Oncol..

[27] O’Leary B, Finn RS, Turner NC (2016). Treating cancer with selective CDK4/6 inhibitors. Nat. Rev. Clin. Oncol..

[28] Goel S (2017). CDK4/6 inhibition triggers anti-tumour immunity. Nature.

[29] Deng J (2018). CDK4/6 inhibition augments antitumor immunity by enhancing T-cell activation. Cancer Discov..

[30] Schaer DA (2018). The CDK4/6 inhibitor abemaciclib induces a T cell inflamed tumor microenvironment and enhances the efficacy of PD-L1 checkpoint blockade. Cell Rep..

[31] Zhang J (2018). Cyclin D-CDK4 kinase destabilizes PD-L1 via cullin 3-SPOP to control cancer immune surveillance. Nature.

[32] Jerby-Arnon L (2018). A cancer cell program promotes T cell exclusion and resistance to checkpoint blockade. Cell.

[33] Bellmunt J, Powles T, Vogelzang NJ (2017). A review on the evolution of PD-1/PD-L1 immunotherapy for bladder cancer: the future is now. Cancer Treat. Rev..

[34] Socinski MA (2018). Atezolizumab for first-line treatment of metastatic nonsquamous NSCLC. N. Engl. J. Med..

[35] Horn S (2018). Tumor CDKN2A-aAssociated JAK2 loss and susceptibility to immunotherapy resistance. J. Natl Cancer Inst..

[36] Helgadottir H (2020). Efficacy of novel immunotherapy regimens in patients with metastatic melanoma with germline CDKN2A mutations. J. Med. Genet..

[37] Frampton GM (2013). Development and validation of a clinical cancer genomic profiling test based on massively parallel DNA sequencing. Nat. Biotechnol..

[38] Pavlick, D. et al. A next-generation sequencing-based karyotyping algorithm reveals the genomic structure of acute myeloid leukemia. *Blood* **132**, 2773 (2018).

[39] Wu TD, Reeder J, Lawrence M, Becker G, Brauer MJ (2016). GMAP and GSNAP for genomic sequence alignment: enhancements to speed, accuracy, and functionality. Methods Mol. Biol..

[40] Friedman J, Hastie T, Tibshirani R (2010). Regularization paths for generalized linear models via coordinate descent. J. Stat. Softw..

[41] Yu G, He QY (2016). ReactomePA: an R/Bioconductor package for reactome pathway analysis and visualization. Mol. Biosyst..

[42] Gu Z, Eils R, Schlesner M (2016). Complex heatmaps reveal patterns and correlations in multidimensional genomic data. Bioinformatics.

