# Supplementary material for: Molecular determinants of response to PD-L1 blockade across tumor types
Source: Nat Commun. 2021 Jun 25;12:3969. doi: 10.1038/s41467-021-24112-w (PMC8233428; doi:10.1038/s41467-021-24112-w)
Supplement: Supplementary file 1 — Supplementary Material [file 41467_2021_24112_MOESM1_ESM.pdf]

Supplementary Figures

Supplementary Figure 1 - Biomarker-evaluable populations summary
Supplementary Figure 2 - Extended PCA in TCGA and PCD4989g and PVCA in Phase II trials RNA-seq datasets
Supplementary Figure 3 - Hierarchical clustering of the correlation matrix of the 61 transcriptional modules identified by WGCNA
Supplementary Figure 4 - Pre-treatment tumor transcriptional phenotypes
Supplementary Figure 5 - Cell population deconvolution by xCell
Supplementary Figure 6 - Benchmarking of machine learning methods
Supplementary Figure 7 - Transcriptional correlates of PD-L1 expression
Supplementary Figure 8 - Transcriptional correlates of tumor mutation burden
Supplementary Figure 9 - Association between ORR and cell populations deconvoluted by xCell
Supplementary Figure 10 - Association between chr9p arm deletions and *CDKN2A* and *CD274* expression

Supplementary Figure 1

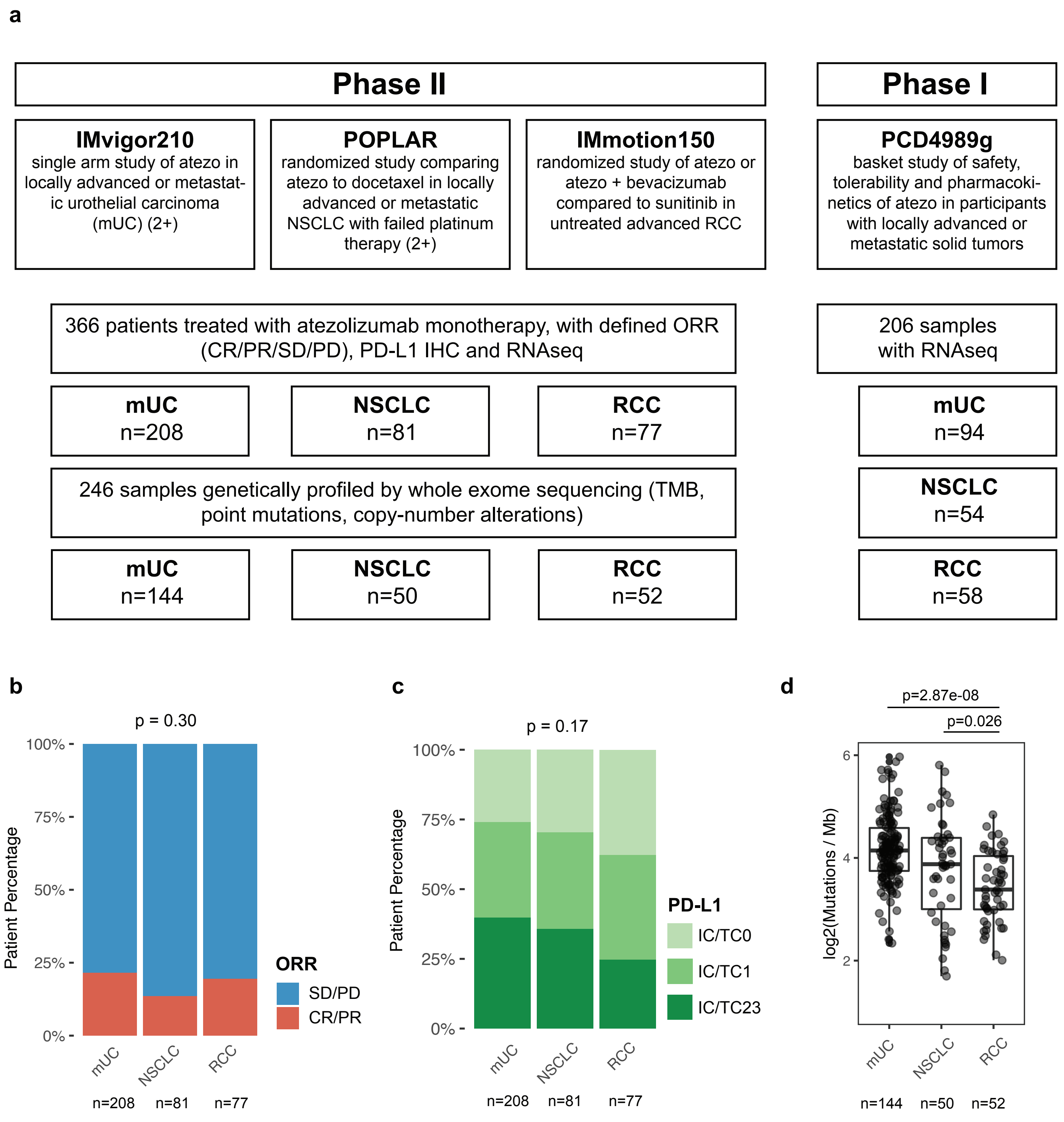

Supplementary Figure 1: Biomarker-evaluable populations summary. a. From three Phase II clinical trials of atezolizumab alone (mUC, IMvigor210) or randomized against docetaxel (NSCLC, POPLAR) or atezolizumab + bevacizumab vs. sunitinib (RCC, IMmotion150), we selected 366 patients treated with atezolizumab monotherapy (mUC: 208; NSCLC: 81; RCC: 77) with defined PD-L1 expression profiles and pre-treatment bulk tumor RNA-seq profiles. Of these, we could also collect tumor whole-exome sequencing data (mUC: 144; NSCLC: 50; RCC: 52). As a validation cohort to identify a transcriptional signature of response to PD-L1 blockade, we selected 206 samples from mUC (n=94), NSCLC (n=54) and RCC (n=58) patients from the basket Phase I clinical trial PCD4989g, whose tumors were profiled for PD-L1 expression and transcription profiles. b. Bar chart representing ORR defined by RECIST v1.1 in the three cohorts. Responders are defined as patients with complete (CR) or partial (PR) responses. Non-responders are defined as patients with stable (SD) or progressive (PD) disease. ORR distribution between indications was statistically tested using the two-sided Pearson's chi-squared test. c. Bar chart representing the distribution of PD-L1 expression on tumor cells (TC) and/or tumor-infiltrating immune cells (IC) by indication. PD-L1 distribution between indications was statistically tested using the two-sided Pearson's chi-squared test. d. Boxplot representing tumor mutation burden (TMB) in the three indications. The middle line represents the median, upper and lower box edges represent the 75th and 25th percentile respectively, whiskers represent the largest/lowest observation less than/greater than or equal upper/lower hinge -/+ 1.5 * inter-quantile range. TMB differences between indications were statistically tested using the non-corrected two-sided Wilcoxon rank sum test.

Supplementary Figure 2

a

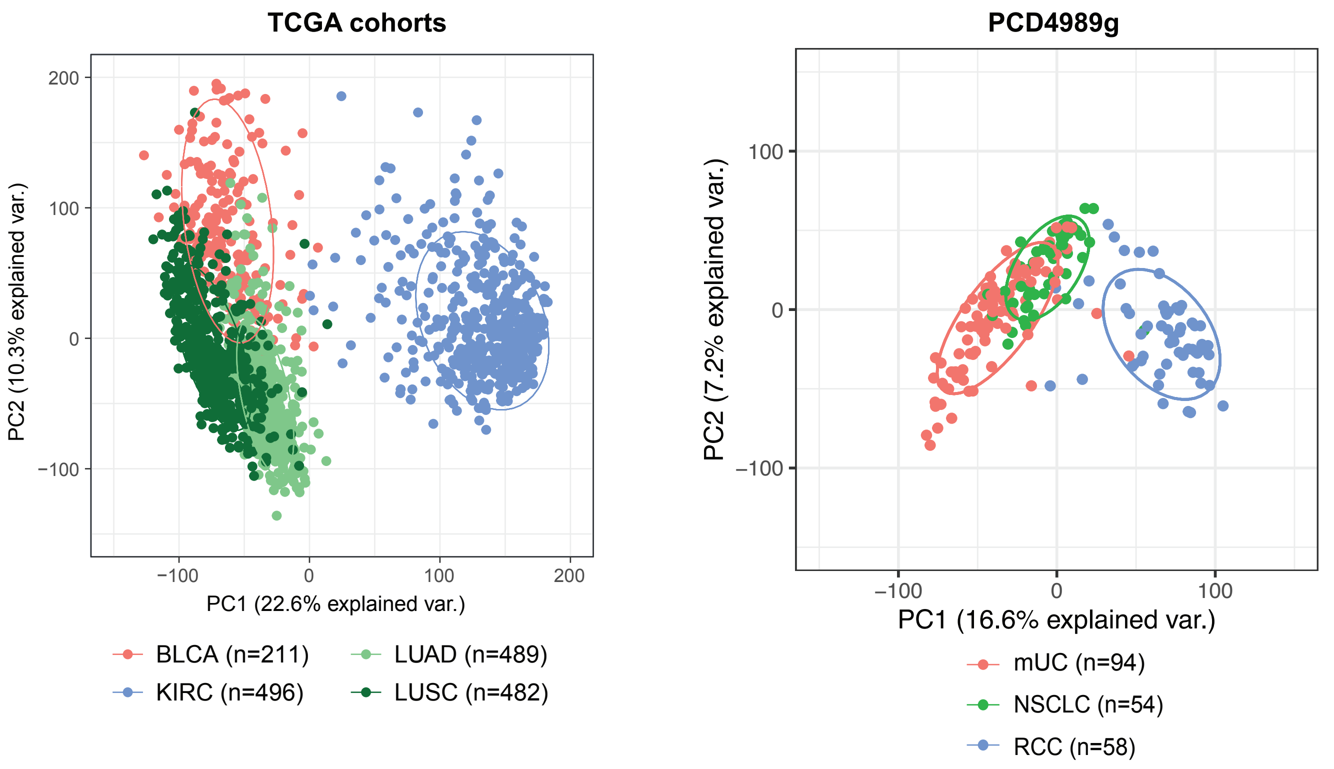

b

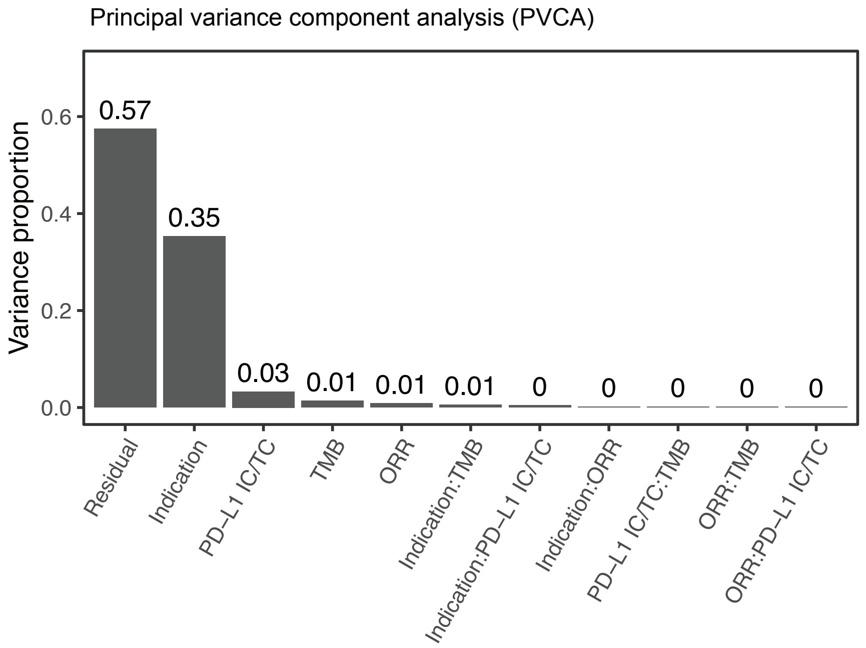

Supplementary Figure 2: Extended PCA in TCGA and PCD4989g and PVCA in Phase II trials RNA-seq datasets. a. Left panel. Principal component analysis (PCA) plot of TCGA samples from bladder cancer (BCLA, n=211), renal clear cell carcinoma (KIRC, n=496), lung adenocarcinoma (LUAD, n=489) and lung squamous cell carcinoma (LUSC, n=482) cohorts, using 15,538 genes overlapping with detected genes from our Phase II mUC, NSCLC and RCC cohorts. Right panel. PCA plot of RNA-seq samples from the mUC (n=121), NSCLC (n=74) and RCC (n=78) cohorts from the atezolizumab monotherapy basket trial PCD4989g, using 16,426 genes. b. Bar chart representing variance proportions quantified by principal variance component analysis. The model tested the contribution of indication, PD-L1 expression, TMB and ORR to the global variance observed in the transcriptional dataset.

Supplementary Figure 3

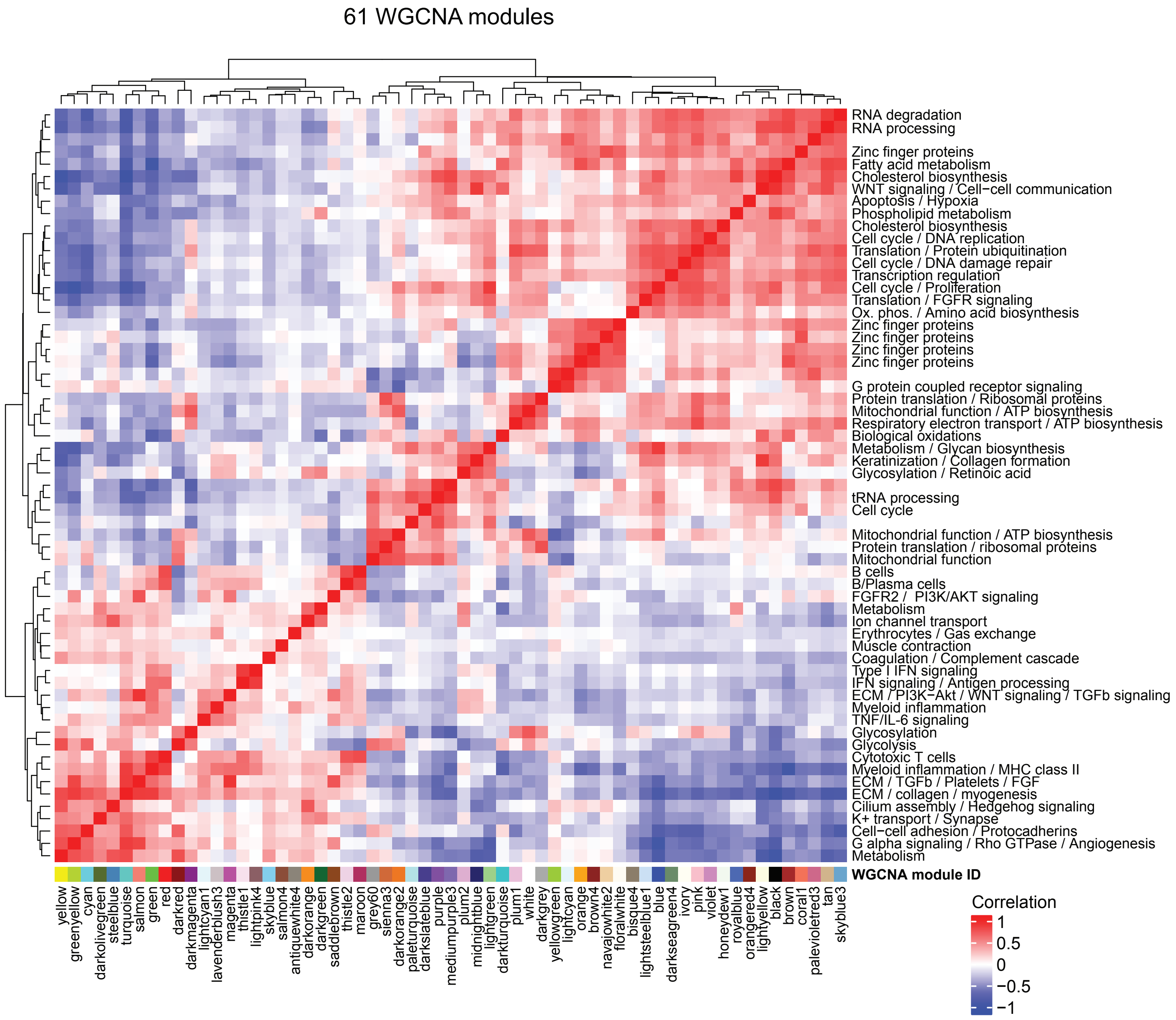

Supplementary Figure 3: Hierarchical clustering of the correlation matrix of the 61 transcriptional modules identified by WGCNA. Module score was calculated for each sample as the mean z-score of the genes composing the signature calculated across the 376 RNA-seq samples. A 61x61 Spearman correlation matrix was obtained using the R cor() function with default settings. The matrix was hierarchically clustered (Euclidian distance) and represented as a heatmap.

Supplementary Figure 4

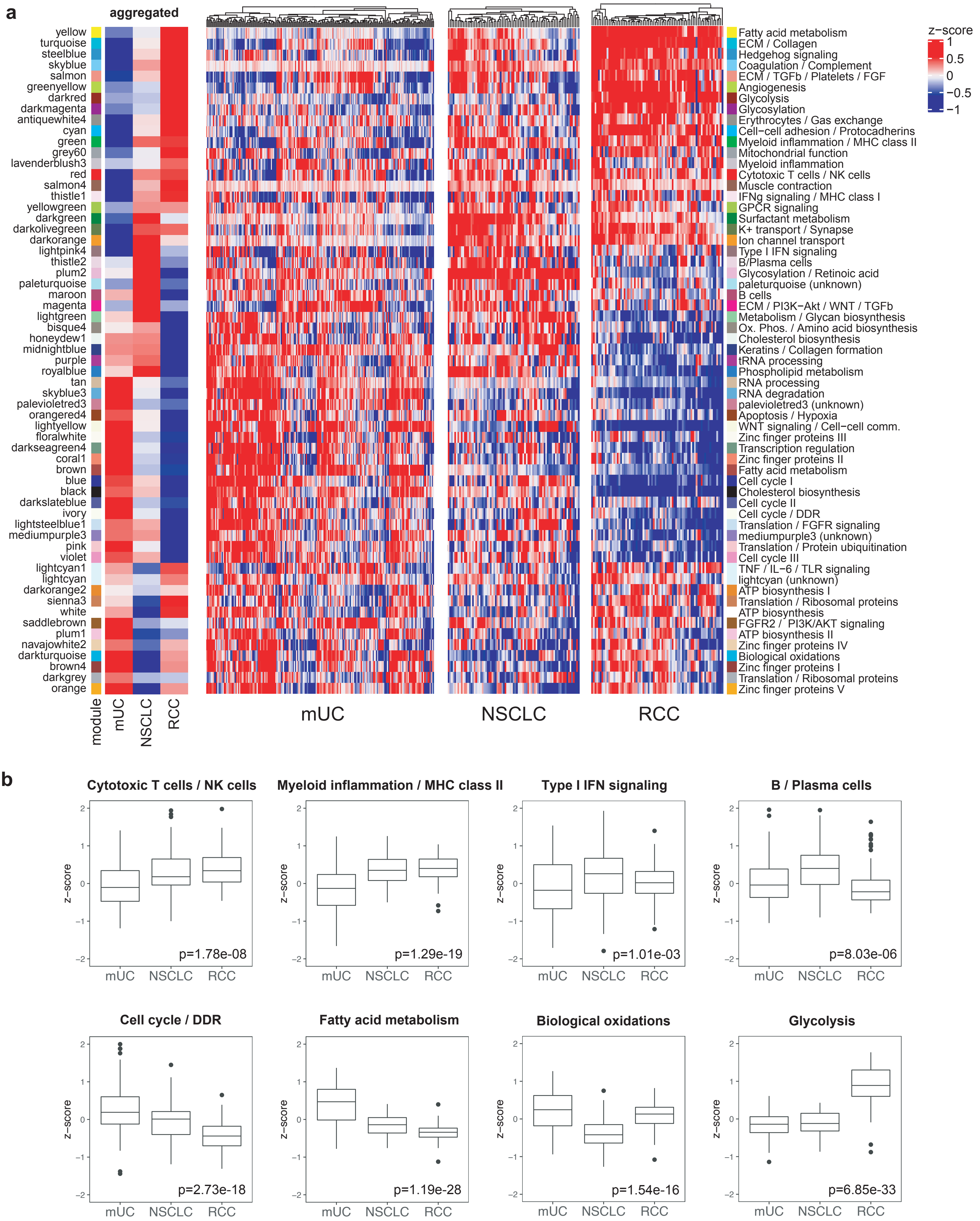

Supplementary Figure 4: Pre-treatment tumor transcriptional phenotypes. a. Heatmap representing the hierarchical clustering (Spearman correlation) of WGCNA modules, aggregated per indication or on an individual patient basis. A module score per indication is obtained by deriving the z-score for each gene across the dataset and computing the median expression of the z-score for genes within a module. Module annotations are obtained as described in Supplemental Materials. b. Boxplots representing the expression of selected immune, angiogenic and proliferative signatures across indications. n=208 mUC, n=81 NSCLC and n=77 RCC biologically independent samples were examined. P-values were calculated using the two-sided Kruskal-Wallis rank sum test. The center of the boxplots represents the median. The lower and upper hinges correspond to the first and third quartiles. The upper whisker extends from the hinge to the largest value no further than 1.5 * IQR (inter-quartile range) from the hinge. The lower whisker extends from the hinge to the smallest value at most 1.5 * IQR of the hinge.

Supplementary Figure 5

a

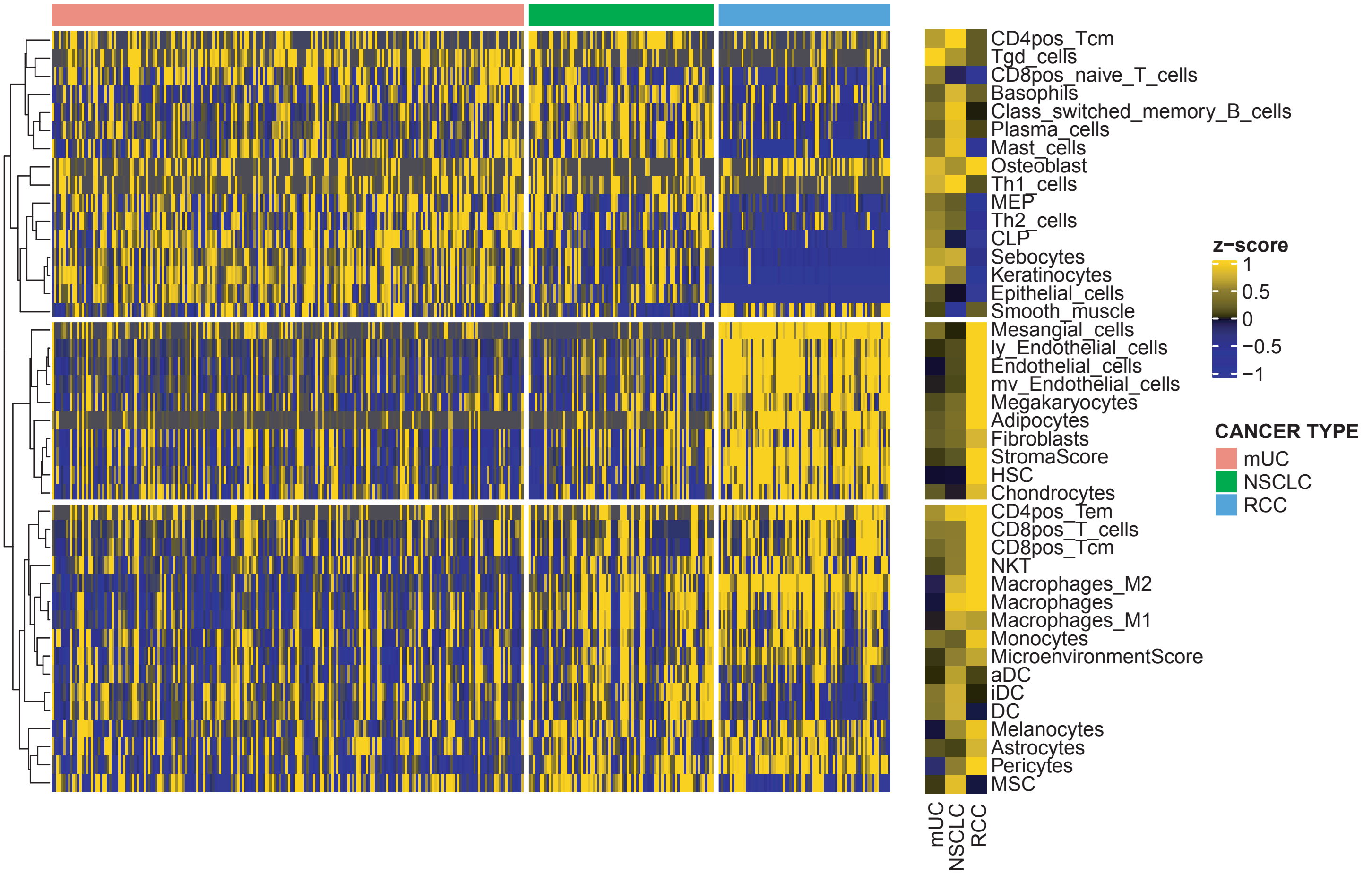

b

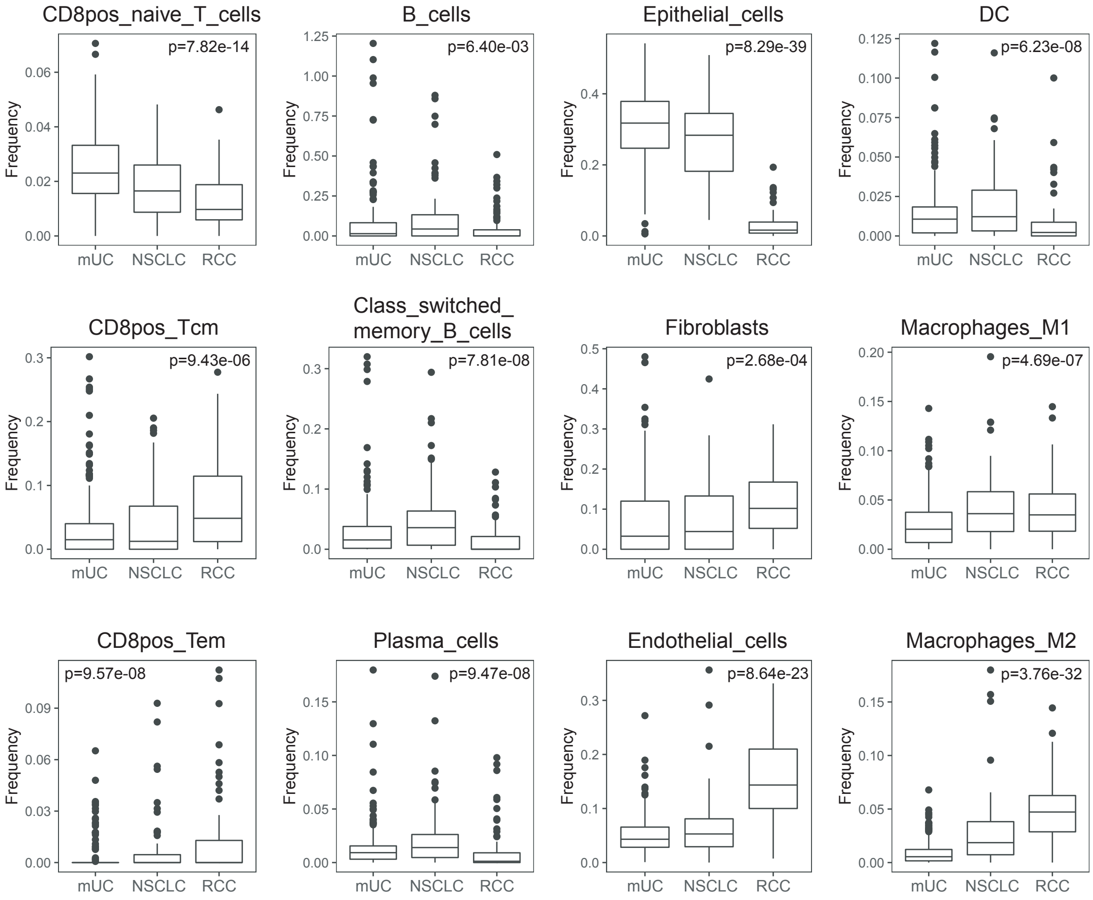

Supplementary Figure 5: Cell population deconvolution by xCell. a. Heatmap of cell populations significantly altered between indications (one-way ANOVA, $p<0.05$). Data are represented as the z-score of population enrichment across the dataset. b. Representative boxplots for data from a., selected for T cell, B cell, myeloid cell and stromal cell components. n=208 mUC, n=81 NSCLC and n=77 RCC biologically independent samples were examined. P-values were calculated using the two-sided Kruskal-Wallis rank sum test. The center of the boxplots represents the median. The lower and upper hinges correspond to the first and third quartiles. The upper whisker extends from the hinge to the largest value no further than 1.5 * IQR (inter-quartile range) from the hinge. The lower whisker extends from the hinge to the smallest value at most 1.5 * IQR of the hinge.

Supplementary Figure 6

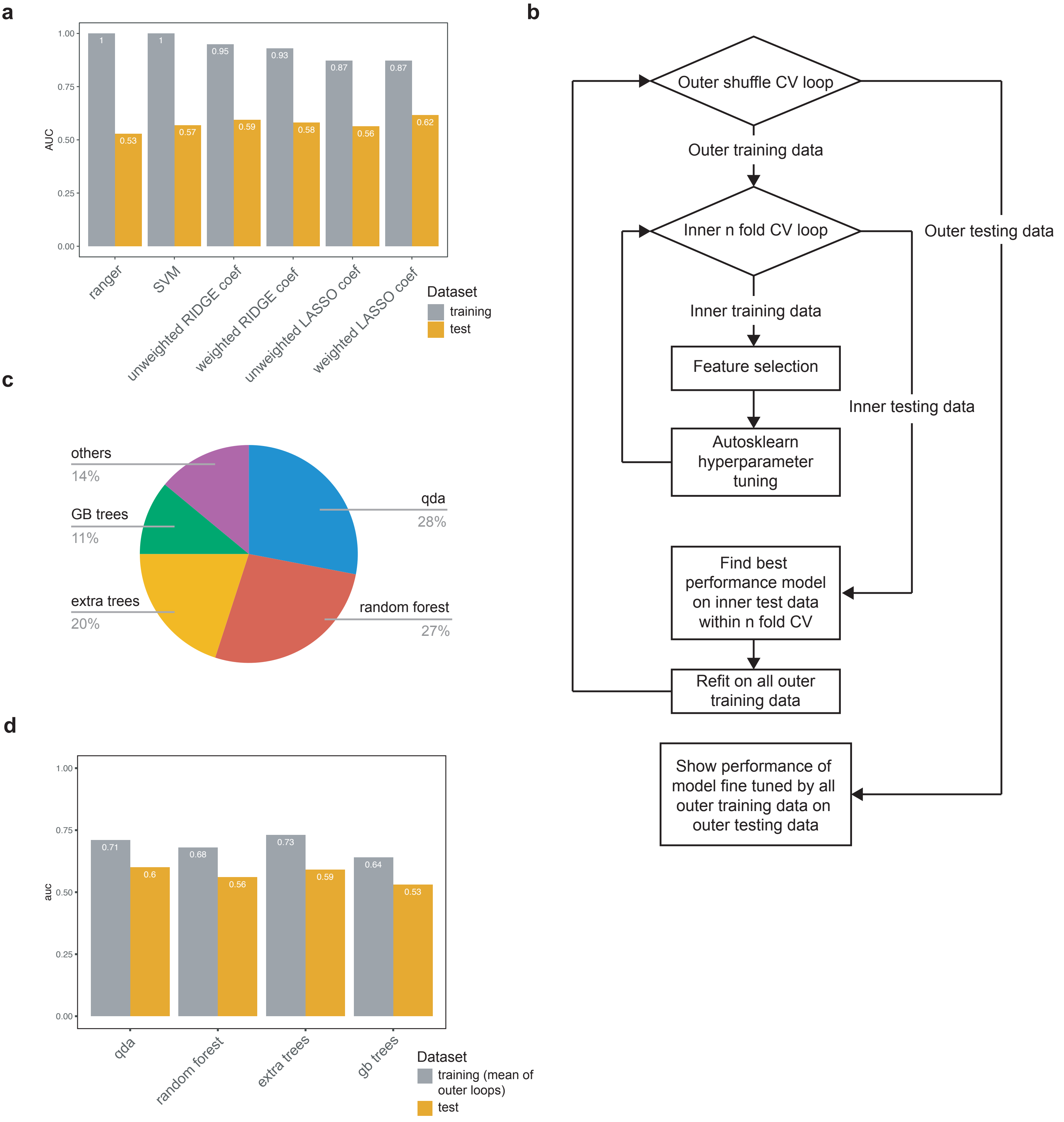

Supplementary Figure 6: Benchmarking of machine learning methods. a. Bar chart representing ROC-AUC results in training and test sets obtained with ranger (random forest), support vector machine (SVM), RIDGE and LASSO (weighted of unweighted). b. Flow chart depicting a nested cross validation approach, using computational tools from the python scikit-learn package. We conducted a stratified shuffled cross validation split method, splitting the whole dataset 100 times into outer training and outer testing datasets in outer loop. The purpose of the outer loop in this design is to prevent data leakage between training and testing and therefore trusting the results in the outer loop as a final independent readout of classification performance. Following data split in the outer loop, the analysis of variance (ANOVA) feature selection method was chosen to reduce the computation complexity from ~16000 genes to 100 genes in the outer training dataset. We chose 100 genes because they included the optimal number of genes for building up all classifiers and they represented a feature set smaller than our patient cohort (n=366). Therefore, we could guarantee that the optimal gene set is within our gene set (top 100) in all classification methods. The purpose of the inner loop was to find the best hyperparameter for each classifier. In this experiment, we used 5-fold cross-validation in the inner loop. The classifiers in the inner loop included all classifiers in the python scikit-learn package. The auto-sklearn package was used to identify the best hyperparameter. We then refit the best classifier with the optimal hyperparameter in the inner loop on all outer training data. Finally, we tested our selected model on the outer testing data. c. Pie chart representing the four models selected the most by the method described in b. d. Bar chart representing the mean ROC-AUC obtained by each model in training and test sets.

Supplementary Figure 7

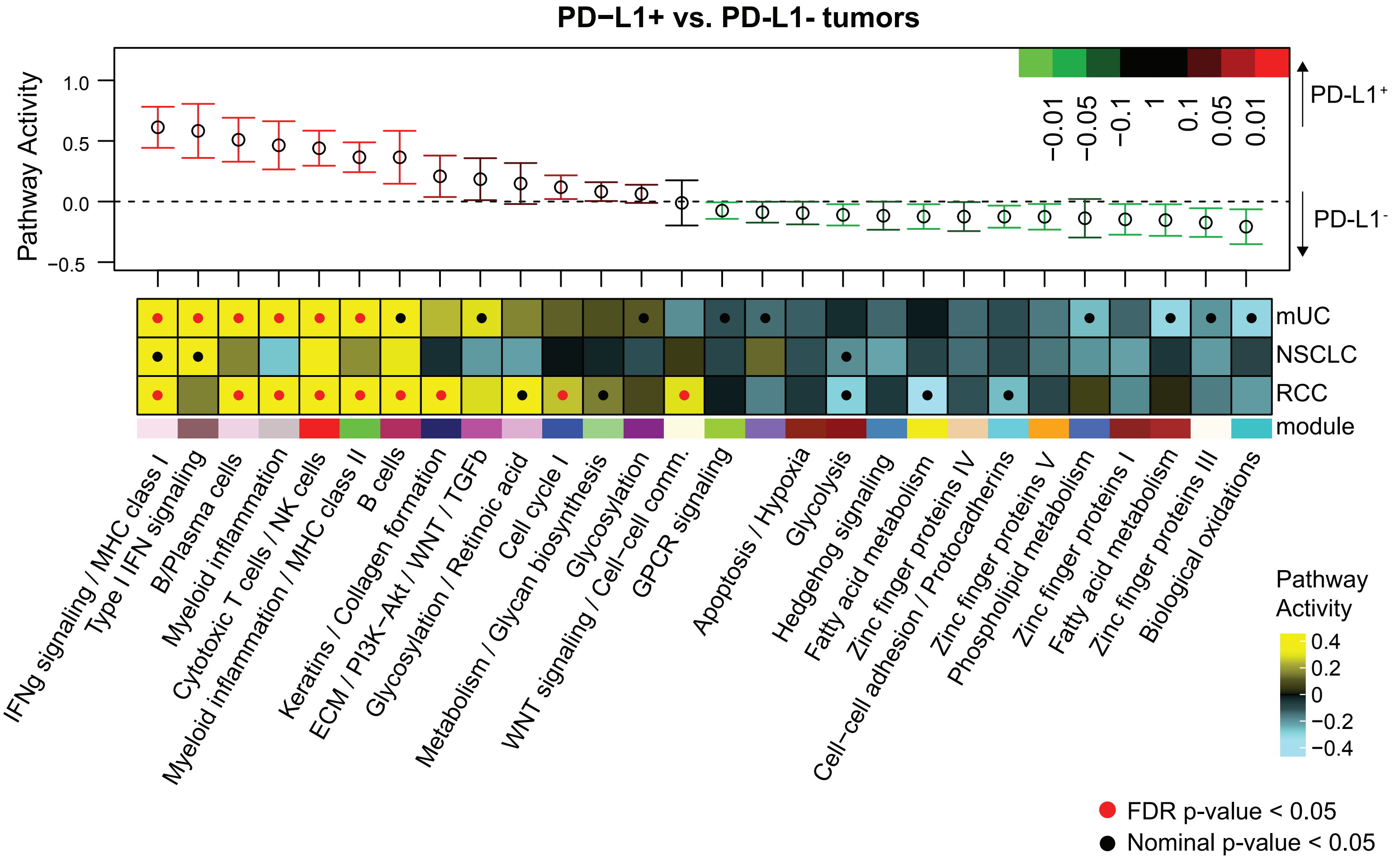

Supplementary Figure 7: Transcriptional correlates of PD-L1 expression. Module-level correlates of PD-L1 expression. The upper panel depicts pathway activity score in the combined cohorts, using indication as a covariate. A high score represents a positive association with PD-L1 expression, a low score a negative association with PD-L1 expression. Error bars represent the 95% confidence interval. The heatmap on the bottom panel represents pathway activity scores when Q-Gen was conducted in each indication separately, highlighting common and specific transcriptional correlates of PD-L1 expression between indications. Modules that exhibited significant pathway activity (nominal p-value < 0.05) either in the combined cohorts or within each cohort separately are represented. Module enrichment significance is highlighted as a red dot (FDR-corrected p<0.05) or a black dot (nominal p<0.05). n=208 mUC, n=81 NSCLC and n=77 RCC biologically independent samples were examined.

Supplementary Figure 8

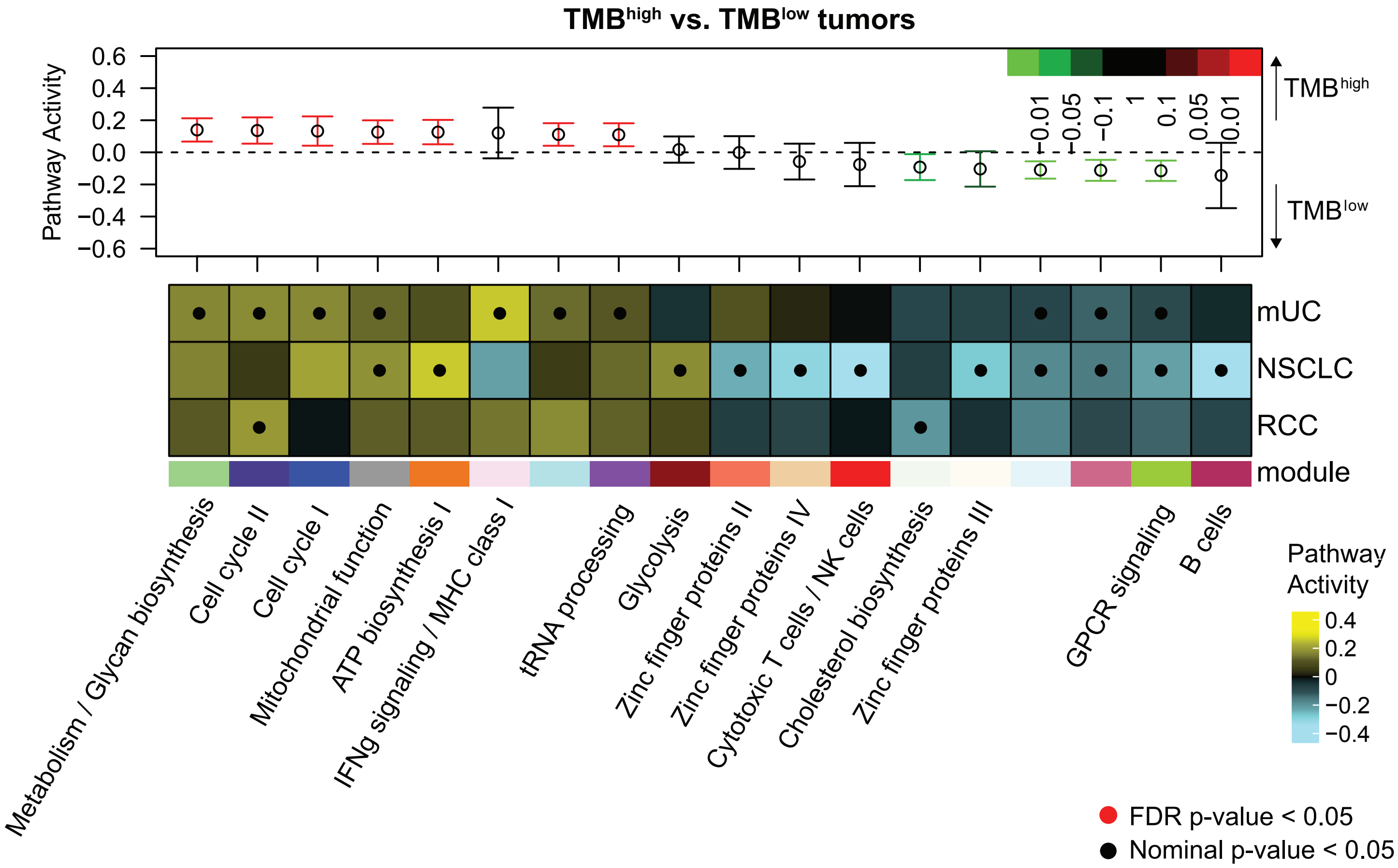

Supplementary Figure 8: Transcriptional correlates of tumor mutation burden. Same as Supplementary Figure 7, testing TMB^{high} vs. TMB^{low} tumors in the Q-Gen contrast. Module enrichment significance is highlighted as a red dot (FDR-corrected $p<0.05$) or a black dot (nominal $p<0.05$). Error bars represent the 95% confidence interval. n=144 mUC, n=50 NSCLC and n=52 RCC biologically independent samples were examined.

Supplementary Figure 9

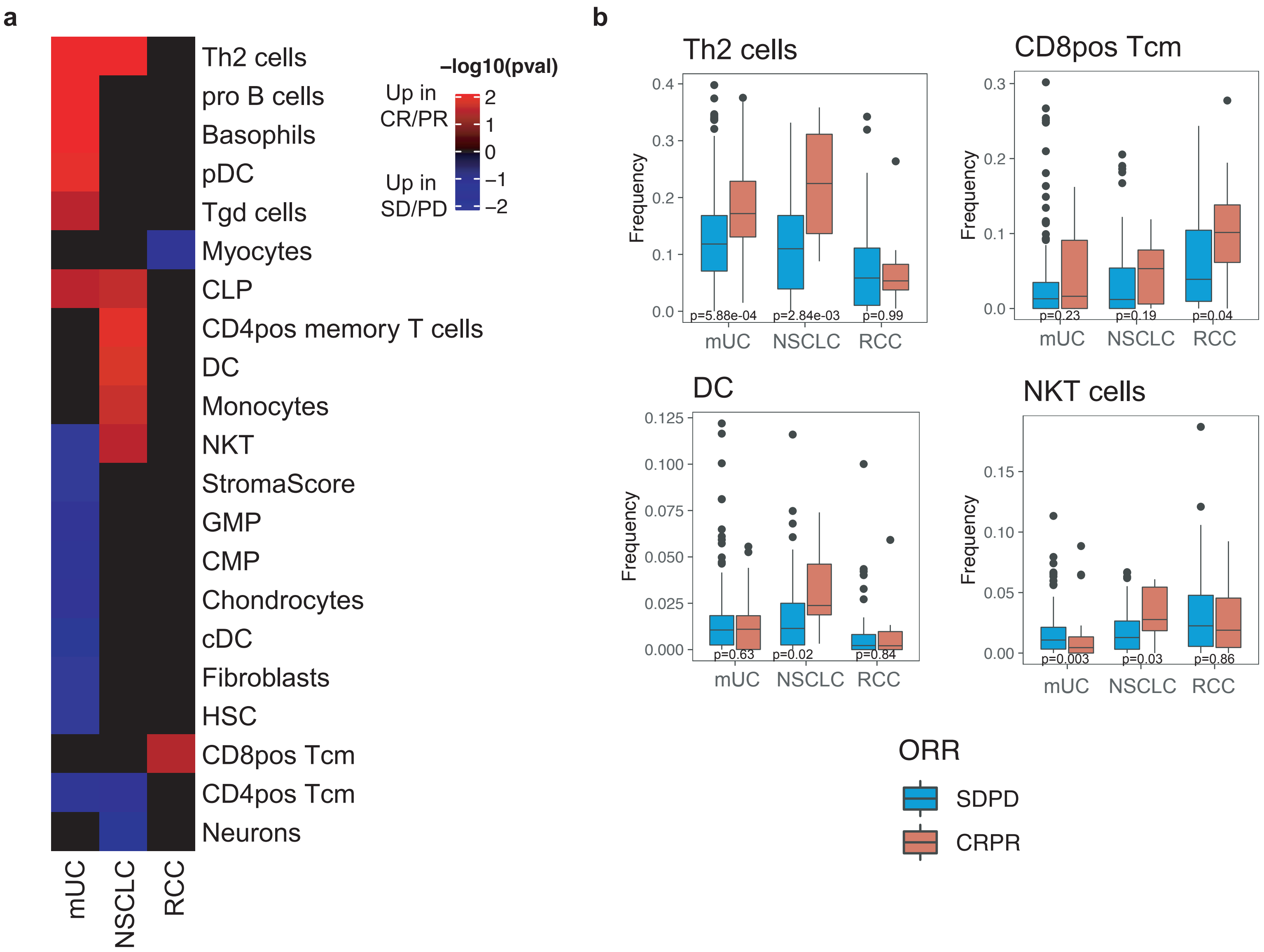

Supplementary Figure 9: Association between cell population deconvolution and ORR. a. Heatmap representing the association between deconvoluted cell population scores and ORR, within aggregated indications. Cells are colored in red or blue if the population deconvolution score is significantly altered between responders and non-responders. Red represents an increase in deconvolution score in responders, blue a decrease. b. Boxplots from representative cell populations from a. n=208 mUC, n=81 NSCLC and n=77 RCC biologically independent samples were examined. P-values were calculated using the two-sided Kruskal-Wallis rank sum test. The center of the boxplots represents the median. The lower and upper hinges correspond to the first and third quartiles. The upper whisker extends from the hinge to the largest value no further than 1.5 * IQR (inter-quartile range) from the hinge. The lower whisker extends from the hinge to the smallest value at most 1.5 * IQR of the hinge.

Supplementary Figure 10

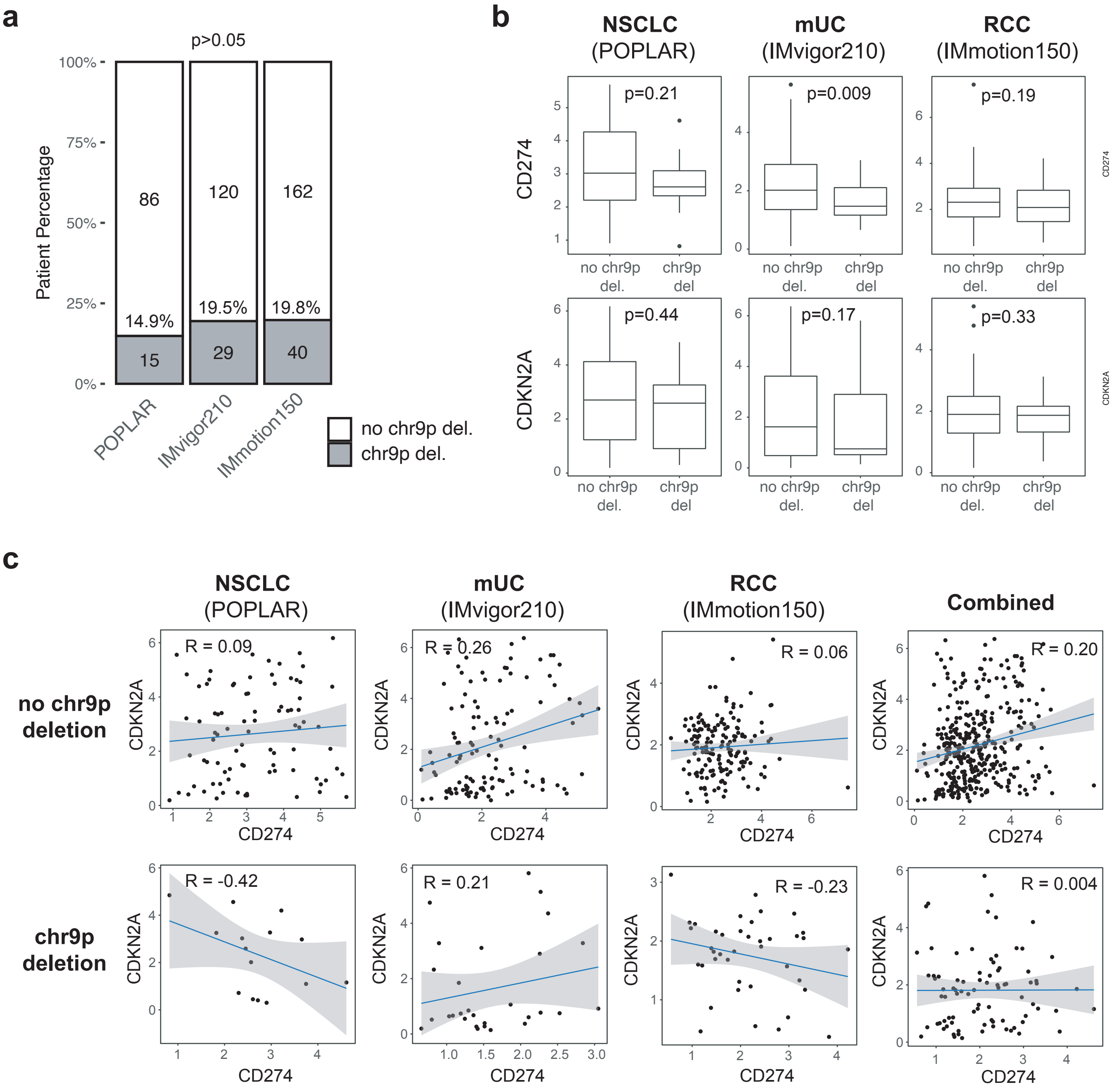

Supplementary Figure 10: Association between chr9p arm deletions and *CDKN2A* and *CD274* expression. a. Bar chart representing the proportion of tumors with shallow chr9p deletions in each indication. Pearson's chi-square test was used to test for differences in distribution. b. Boxplots representing the log2(TPM) expression of *CD274* (encoding PD-L1) and *CDKN2A* in tumors with or without chr9p arm deletions within each indication or combined. The two-sided Wilcoxon rank sum test was used to compare groups. n=149 mUC, n=101 NSCLC and n=202 RCC biologically independent samples were examined. The center of the boxplots represents the median. The lower and upper hinges correspond to the first and third quartiles. The upper whisker extends from the hinge to the largest value no further than 1.5 * IQR (inter-quartile range) from the hinge. The lower whisker extends from the hinge to the smallest value at most 1.5 * IQR of the hinge. c. X-Y charts representing the correlation between *CD274* and *CDKN2A* expression in tumors with or without chr9p arm deletions, within each indication or combined. The two-sided Pearson R correlation coefficient was reported. The error bands represent the 95% percentile of the linear fit.
