# Supplementary material for: Molecular determinants of response to PD-L1 blockade across tumor types
Source: Nat Commun. 2021 Jun 25;12:3969. doi: 10.1038/s41467-021-24112-w (PMC8233428; doi:10.1038/s41467-021-24112-w)
Supplement: Supplementary file 2 — Descriptions of Additional Supplementary Files [file 41467_2021_24112_MOESM2_ESM.pdf]

Descriptions of Additional Supplementary Files

Supplementary Data 1
Description: Patient characteristics.

Supplementary Data 2
Description: 58-gene signature identified by LASSO.

Supplementary Data 3
Description: Transcriptional correlates of PD-L1 expression.

Supplementary Data 4
Description: Transcriptional correlates of TMB.

Supplementary Data 5
Description: Transcriptional correlates of ORR.

Supplementary Data 6
Description: Module correlation with TMB
