# Supplementary material for: Molecular determinants of response to PD-L1 blockade across tumor types
Source: Nat Commun. 2021 Jun 25;12:3969. doi: 10.1038/s41467-021-24112-w (PMC8233428; doi:10.1038/s41467-021-24112-w)
Supplement: Supplementary file 9 — Reporting Summary [file 41467_2021_24112_MOESM9_ESM.pdf]

nature research

Corresponding author(s): Thomas Powles
Romain Banchereau

Last updated by author(s): Mar 30, 2021

Reporting Summary

Nature Research wishes to improve the reproducibility of the work that we publish. This form provides structure for consistency and transparency in reporting. For further information on Nature Research policies, see Authors & Referees and the Editorial Policy Checklist .

Statistics

For all statistical analyses, confirm that the following items are present in the figure legend, table legend, main text, or Methods section.

n/a	Confirmed	
☐	☒	The exact sample size (*n*) for each experimental group/condition, given as a discrete number and unit of measurement
☐	☒	A statement on whether measurements were taken from distinct samples or whether the same sample was measured repeatedly
☐	☒	The statistical test(s) used AND whether they are one- or two-sided<br>*Only common tests should be described solely by name; describe more complex techniques in the Methods section.*
☐	☒	A description of all covariates tested
☐	☒	A description of any assumptions or corrections, such as tests of normality and adjustment for multiple comparisons
☐	☒	A full description of the statistical parameters including central tendency (e.g. means) or other basic estimates (e.g. regression coefficient) AND variation (e.g. standard deviation) or associated estimates of uncertainty (e.g. confidence intervals)
☐	☒	For null hypothesis testing, the test statistic (e.g. *F*, *t*, *r*) with confidence intervals, effect sizes, degrees of freedom and *P* value noted<br>*Give P values as exact values whenever suitable.*
☒	☐	For Bayesian analysis, information on the choice of priors and Markov chain Monte Carlo settings
☒	☐	For hierarchical and complex designs, identification of the appropriate level for tests and full reporting of outcomes
☒	☐	Estimates of effect sizes (e.g. Cohen's *d*, Pearson's *r*), indicating how they were calculated

Our web collection on statistics for biologists contains articles on many of the points above.

Software and code

Policy information about availability of computer code

Data collection	The data presented in this manuscript are available in EGA. Clinical data was collected and organized with SAS and R.
Data analysis	All R/Bioconductor packages used for data analysis were cited in Methods, along with the version used. Specialized packages include Sequenza version 2.2.0.9000, pvca v1.18.0., glmnet v2.0-13., limma 3.46.0, WGCNA 1.64-1, ReactomePA 1.22.0, ComplexHeatmap 2.6.2, QuSAGE 2.12.0. The code necessary for reproducing the figures is also shared.

For manuscripts utilizing custom algorithms or software that are central to the research but not yet described in published literature, software must be made available to editors/reviewers. We strongly encourage code deposition in a community repository (e.g. GitHub). See the Nature Research guidelines for submitting code & software for further information.

Data

Policy information about availability of data

All manuscripts must include a data availability statement. This statement should provide the following information, where applicable:

- Accession codes, unique identifiers, or web links for publicly available datasets
- A list of figures that have associated raw data
- A description of any restrictions on data availability

All raw RNA-seq and whole exome sequencing data, along with clinical data, are deposited to the European Genome-Phenome Archive under accession number EGAS00001004343. There are not restrictions on data availability.

Field-specific reporting

Please select the one below that is the best fit for your research. If you are not sure, read the appropriate sections before making your selection.

Life sciences study design

All studies must disclose on these points even when the disclosure is negative.

Sample size	No sample size calculation was conducted. These restrospective biomarker studies were conducted in patients from 3 phase II studies where tumor tissue was available. These patients all had defined ORR (CR/PR/SD/PD), defined PD-L1 status by IHC and baseline bulk RNA-seq profiles. Not all patients have TMB data, so numbers vary based on the biomarker evaluable populations.
Data exclusions	Because our study was focused on transcriptional programs associated with objective response rate, defined as CR, PR, SD or PD, patients with undefined ORR were excluded from analysis.
Replication	We tested our gene signature classifier in an independent cohort of 206 patients that included NSCLC, mUC and RCC tumors. The performance of our signature in this specific test set was poor.
Randomization	IMvigor210 was a single arm phase II clinical trial of atezolizumab in mUC. For trials were other arms were available (including POPLAR and IMmotion150), patients were randomized 1:1 to atezolizumab vs. docetaxel or sunitinib respectively.
Blinding	Full description of the study protocols and blinding are detailed in the following publications:<br>IMvigor210: Balar AV, et al. Lancet, 2017;389(10064):67-76.<br>POPLAR: Fehrenbacher L, et al. Lancet, 2016 Apr 30;387(10030):1837-46.<br>IMmotion150: McDermott D, et al. Nat Med. 2018 Jun;24(6):749-757.<br>These papers have been referenced in this manuscript.

Reporting for specific materials, systems and methods

We require information from authors about some types of materials, experimental systems and methods used in many studies. Here, indicate whether each material, system or method listed is relevant to your study. If you are not sure if a list item applies to your research, read the appropriate section before selecting a response.

Materials & experimental systems

n/a	Involved in the study	
☐	☒	Antibodies
☒	☐	Eukaryotic cell lines
☒	☐	Palaeontology
☒	☐	Animals and other organisms
☐	☒	Human research participants
☐	☒	Clinical data

Methods

n/a	Involved in the study	
☒	☐	ChIP-seq
☒	☐	Flow cytometry
☒	☐	MRI-based neuroimaging

Antibodies

Antibodies used	anti-PD-L1 SP142, Ventana
Validation	The package insert is available at https://www.accessdata.fda.gov/cdrh_docs/pdf16/P160002c.pdf

Human research participants

Policy information about studies involving human research participants

Population characteristics	We are presenting data from patients from 3 indications, including mUC, NSCLC and RCC, with various levels of PD-L1 expression on immune or tumor cells. Both indication and PD-L1 expression were used in our linear models. 75.1% were male (275/366). The median age was 61.5 [range 26-82].
Recruitment	Full description of the human research participants and recruitment are detailed in the following publications:<br>IMvigor210: Balar AV, et al. Lancet, 2017;389(10064):67-76.<br>POPLAR: Fehrenbacher L, et al. Lancet, 2016 Apr 30;387(10030):1837-46.<br>IMmotion150: McDermott D, et al. Nat Med. 2018 Jun;24(6):749-757.<br>These papers have been referenced in this manuscript.
Ethics oversight	We complied with all relevant ethical regulations for work with human participants, and informed consent was obtained from all patients. The protocol was approved by the institutional review boards or independent ethics committees at each participating center.

Note that full information on the approval of the study protocol must also be provided in the manuscript.

Clinical data

Policy information about clinical studies

All manuscripts should comply with the ICMJE guidelines for publication of clinical research and a completed CONSORT checklist must be included with all submissions.

Clinical trial registration	Data from 3 trials are presented in this manuscript. IMvigor210: NCT02108652; POPLAR: NCT01903993; IMmotion150: NCT01984242
Study protocol	The studies have reported and the full protocols are availabe on clinicaltrials.gov
Data collection	Full description of the human research participants and clinical data collection are detailed in the following publications:<br>IMvigor210: Balar AV, et al. Lancet, 2017;389(10064):67-76.<br>POPLAR: Fehrenbacher L, et al. Lancet, 2016 Apr 30;387(10030):1837-46.<br>IMmotion150: McDermott D, et al. Nat Med. 2018 Jun;24(6):749-757.<br>These papers have been referenced in this manuscript.
Outcomes	Full description of the human research participants and clinical outcomes are detailed in the following publications:<br>IMvigor210: Balar AV, et al. Lancet, 2017;389(10064):67-76.<br>POPLAR: Fehrenbacher L, et al. Lancet, 2016 Apr 30;387(10030):1837-46.<br>IMmotion150: McDermott D, et al. Nat Med. 2018 Jun;24(6):749-757.<br>These papers have been referenced in this manuscript.
